# Implicit Visuomotor Adaptation Remains Limited after Several Days of Training

**DOI:** 10.1523/ENEURO.0312-20.2021

**Published:** 2021-08-10

**Authors:** Sarah A. Wilterson, Jordan A. Taylor

**Affiliations:** Department of Psychology, Princeton Neuroscience Institute, Princeton University, Princeton, NJ 08544

**Keywords:** adaptation, explicit, implicit, motor, reaching, strategy

## Abstract

Learning in sensorimotor adaptation tasks has been viewed as an implicit learning phenomenon. The implicit process affords recalibration of existing motor skills so that the system can adjust to changes in the body or environment without relearning from scratch. However, recent findings suggest that the implicit process is heavily constrained, calling into question its utility in motor learning and the theoretical framework of sensorimotor adaptation paradigms. These inferences have been based mainly on results from single bouts of training, where explicit compensation strategies, such as explicitly re-aiming the intended movement direction, contribute a significant proportion of adaptive learning. It is possible, however, that the implicit process supersedes explicit compensation strategies over repeated practice sessions. We tested this by dissociating the contributions of explicit re-aiming strategies and the implicit process in human participants over five consecutive days of training. Despite a substantially longer duration of training, the implicit process still plateaued at a value far short of complete learning and, as has been observed in previous studies, was inappropriate for a mirror-reversal task. Notably, we find significant between subject differences that call into question traditional interpretation of these group-level results.

## Significance Statement

In this set of studies, we find that the implicit process cannot fully account for learning in adaptation tasks, such as the visuomotor rotation and mirror-reversal tasks, even following several days of training. In fact, the implicit process can be counterproductive to learning. Most notably, we find significant between subject differences that call into question traditional interpretation of these group-level results.

## Introduction

A significant perturbation must be overcome when using a computer mouse to navigate around a monitor screen. If sliding the hand forward moves the cursor up the screen, a 90° rotation has been applied to the visual feedback of the hand. Despite this discrepancy, we do not feel as though we are explicitly compensating for the shift between hand and computer mouse. The movement is made automatically, with little conscious effort necessary to achieve the desired goal. Intuitively, this suggests an important role for the implicit process, compensation without strategic intervention, in motor skill. Recent research has focused on characterizing the mechanisms giving rise to the implicit processes used in motor skill learning, mostly through sensorimotor adaptation paradigms. A common approach is to ask participants to make point-to-point reaching movements to nearby targets while the hand is obscured from view and visual (or proprioceptive) feedback is artificially perturbed (Held and Schlank, 1959; Cunningham, 1989; Imamizu et al., 1995; Pine and Krakauer et al., 1996; Krakauer, 2009).

The results of these sensorimotor adaptation experiments have been interpreted under the framework that the implicit process is driving a significant proportion of learning (Scheidt et al., 2001; Baddeley et al., 2003; Fine and Thoroughman, 2006, 2007; Wei and Körding, 2009). However, a number of more recent studies have challenged this view. Initially, these studies disassociated explicit compensation strategies and the implicit process with postexperimental assays and questionnaires (Heuer and Hegele, 2008; Hegele and Heuer, 2010), instruction (Mazzoni and Krakauer, 2006; Benson et al., 2011), and modeling (Taylor and Ivry, 2011). This important early work showed that the slow, gradual learning of the implicit process is not the only process at play in a visuomotor rotation task.

In parallel to the recognition that strategies play an important role in adaptation tasks, we see rapidly emerging evidence that the implicit process is highly stereotyped regardless of the particular task demands. The implicit process asymptotes to a value far less than complete learning and is largely insensitive to perturbation magnitude (Bond and Taylor, 2015; Morehead et al., 2017). Morehead and colleagues revealed a stereotyped implicit learning curve when the incentive to strategize was completely removed. Surprisingly, asymptotic implicit learning remained insufficient to account for the perturbation after many hours of practice (Morehead and Smith, 2017; Morehead et al., 2017; Kim et al., 2018). Finally, recent evidence suggests that the contribution of the implicit process decreases with extended training (Avraham et al., 2021).

The essential role of the implicit process may be in fine adjustments to very small errors. A few recent reports have shown that the implicit process is proportional for rotations under ∼8° and is only slightly affected by task constraints, such as reward (Kim et al., 2018, 2019; Leow et al., 2018; Hutter and Taylor, 2018). Given all of this, it is difficult to see the role of an asymptotically constrained implicit process in long-term motor learning.

However, a rest period, following exposure to a perturbed training environment, allows for continued learning in the absence of the stimuli (Brashers-Krug et al., 1996). If a significant rest period is needed for the consolidation of learning, a single session may be insufficient to determine how the implicit process contributes over the long-term. The full effect of adaptation seen by Stratton (1896, 1897) and Kohler (1941, 1951a,b), complete adaptation to an inverted world without need for explicit compensation, occurred following five full days of continuous exposure. Implicit learning may require extensive consolidation periods to fully compensate for a large visuomotor rotation (Krakauer and Shadmehr, 2006; Criscimagna-Hemminger and Shadmehr, 2008; but see Caithness et al., 2004).

In experiment 1, we investigate adaptative learning to a visual perturbation over 5 d of training. If the implicit process, as measured in this task, eventually contributes to the majority of overall compensation, we expect to see a steady increase in the proportion of learning accounted for by the implicit process over time. We find that the implicit process, on average, does not change over the training period. However, we also find substantial between-subject differences that raise questions about the validity of the measure.

**Figure 1.**
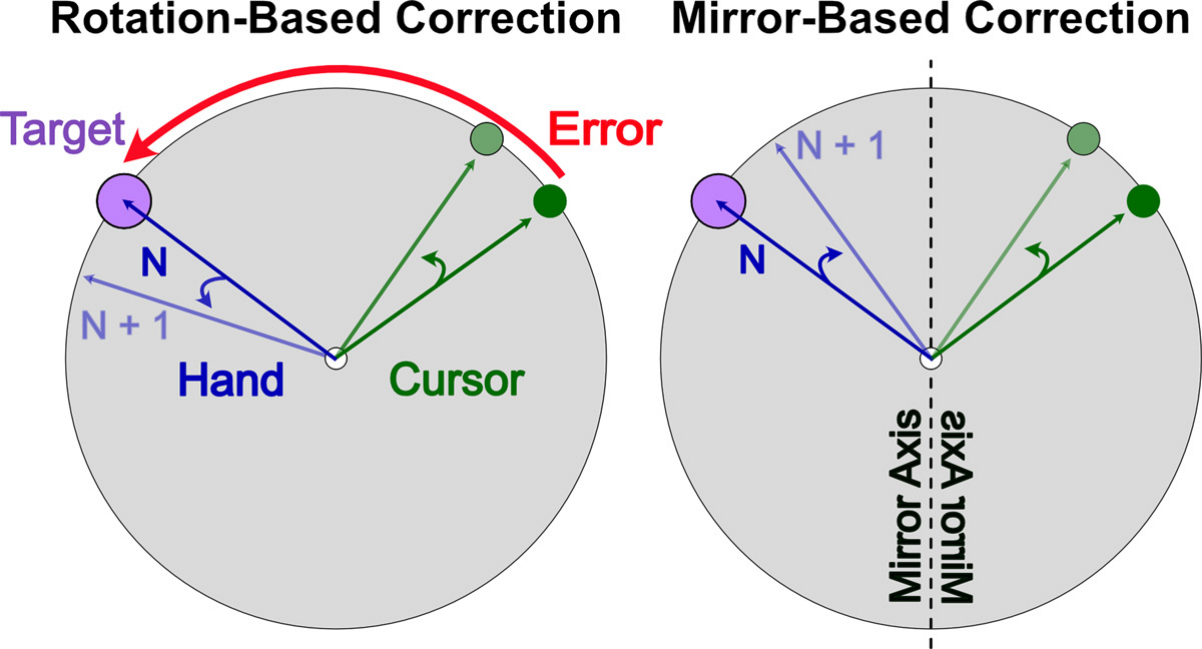
Appropriate correction for both rotation and mirror-reversal perturbations. If the goal is to reduce error between the cursor and the target, the adaptive correction, denoted by the blue arrow, is counterclockwise for the rotation-based correction but clockwise for the mirror-based correction. Prior evidence supports the implicit process being appropriate for the rotation but not for the mirror. This has been used as evidence that visuomotor rotation tasks reveal adaptation of an established skill (reaching), while mirror-reversal tasks reveal a *de novo* skill that does not benefit from adaptation.

An additional complication, not considered in experiment 1, is the directional nature of the implicit process. The implicit response to directional information appears to be automatic: the motor system adapts in a direction opposite to the perturbation even when such adaptation is task-irrelevant (Schaefer et al., 2012; Morehead et al., 2017; Butcher and Taylor, 2018). This response to directional information has led researchers to question the role of the implicit process in *de novo* skill learning, such as the visuomotor mirror-reversal task. This task requires a directional adaptation response opposite to that seen in rotation tasks (Fig. 1; Krakauer, 2009; Gritsenko and Kalaska, 2010; Lillicrap et al., 2013; Telgen et al., 2014).

Indeed, there is evidence that, even after a few days of training, the implicit process works against performance in mirror-reversal tasks (Telgen et al., 2014; Kasuga et al., 2015). However, complicating this picture, a mirror-reversal task is exactly what bilateral hippocampal lesion patient H.M. was able to complete, presumably without the benefit of explicit processes (Corkin, 1968). How was this accomplished if the implicit process, as we know it from previous visuomotor rotation studies, is unable to solve a mirror reversal? In experiment 2, we sought to determine whether sufficient practice would transform the mirror-reversal task from one of *de novo* learning, to an adaptation task. That is, if the implicit process could become useful in a mirror task given a sufficiently long training period. This would give deeper insight into the role of the implicit process in early and long-term learning of novel motor skills. We found that, on average, the implicit process gradually decreases over time, slowly becoming more appropriate for the mirror-reversal task. However, we again find substantial between-subject differences.

## Materials and Methods

In two experiments, 31 subjects participated in either a visuomotor rotation task or mirror-reversal task for 1 h each day for five consecutive days. Subjects attempted to compensate for a visual perturbation of 45° while reaching to targets that appeared on a circle centered on the start position.

### Participants

Seventeen first year graduate students, undergraduate students, and community members were recruited to participate in experiment 1. Of the seventeen, three subjects were removed from the experiment because of equipment failure and two additional subjects dropped out of the experiment without completing all 5 d. The remaining 12 subjects (five female, age 24.04 ± 2.11 years) successfully completed the full experiment and their data are reported here. To have an equivalent number of subjects in the second experiment, fourteen subjects were recruited in the same manner as in experiment 1. Two of these subjects retired from the experiment before completion. Data are presented for the remaining 12 subjects (eight female, age 23.76 ± 3.04 years).

Subjects were verified to be right handed by the Edinburgh Handedness Inventory (Oldfield, 1971) and self-reported having normal or corrected-to-normal vision. The experimental protocol was approved by the Princeton University Institutional Review Board and all subjects provided written, informed consent. All subjects received monetary compensation for their participation.

We did not have an a priori effect size estimate with which to calculate a power analysis. However, assuming a 3° change in mean and a SD of 3° results in a suggested sample of 10 participants to achieve 90% power. A *post hoc* power analysis conducted on the reduction of implicit adaptation found in experiment 2 results in a power level of just over 85% to observe the reported effect.

### Apparatus

**Figure 2.**
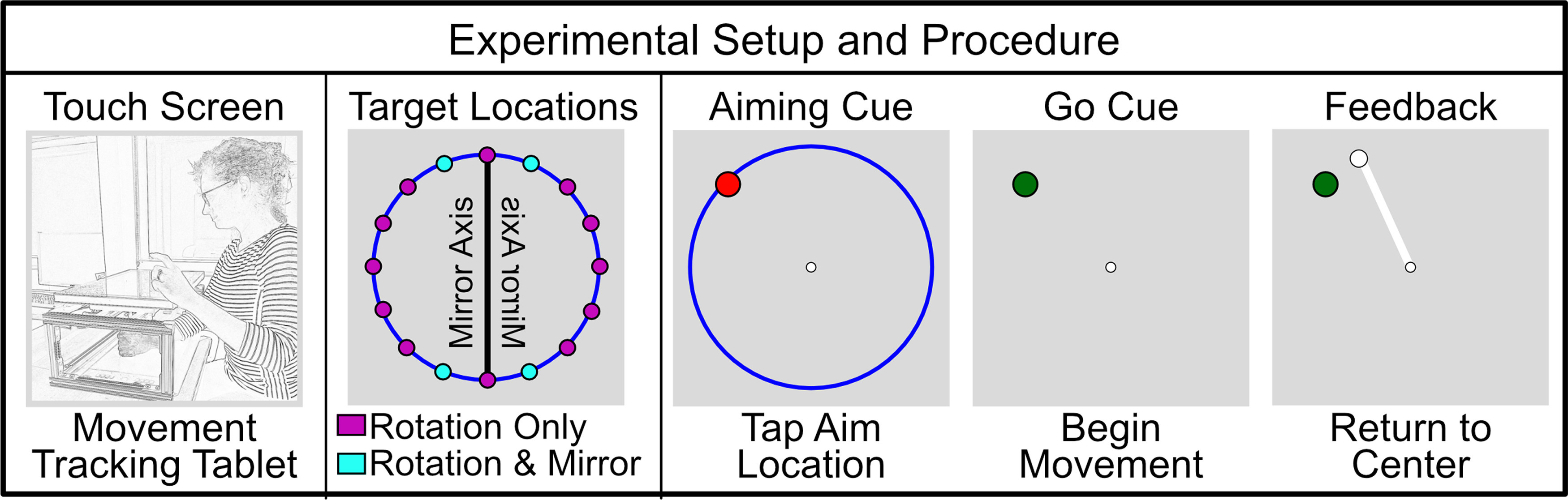
Experimental paradigm for assaying the implicit process to an imposed visual perturbation. The target locations for the mirror task were chosen to maintain a 45° perturbation (±22.5° from the mirror axis). Participants indicate their intended movement direction (re-aiming) by tapping on the aiming ring, which is shown here in blue. They then use the right hand to reach for the location that they think will bring the cursor to the target (ostensibly the re-aimed location). Any learning not accounted for by explicit re-aiming is presumed to be the implicit process.

Subjects performed horizontal movements in a center-out reaching task similar to that first described in Bond and Taylor (2017; Fig. 2). Stimuli were displayed on a 60 Hz, 17-in., Planar touch sensitive monitor (Planar Systems) and computed by a Dell OptiPlex 7040 machine (Dell) running Windows 7 (Microsoft Co). Movements were recorded with a Wacom magnetic digitizing pen and tablet (Wacom Co). Aiming locations were recorded by tapping the touch-sensitive monitor, which was placed 25 cm above the Wacom tablet and obscured visual access to the right hand. The game was controlled by custom software coded in MATLAB (The MathWorks), using Psychtoolbox extensions (Brainard and Vision, 1997; Kleiner et al., 2007).

### Procedure

In order to assay the operation of explicit strategies and the implicit process on a trial-by-trial basis, we asked participants to report their intended aiming location (i.e., the direction that they intend to move to compensate for the perturbation) by tapping the surface of a touch screen (following Bond and Taylor, 2017; Hutter and Taylor, 2018). Participants reported their intended aiming location and then made a reaching movement. The difference between the intended movement (explicit re-aiming) and the actual reach direction reveals any learning of which the subject was unaware (the implicit process).

Each trial began with subjects holding their right hand in the center of the workspace for 300 ms. Then, a circular orange target (0.25-cm radius) appeared 7 cm from the start position on a blue, 7 cm radius “aiming ring.” To begin a trial, the subject indicated their intended reach direction by tapping on the aiming ring with their left hand. Once an aim was recorded, the target turned from orange to green, the aiming ring disappeared, and the subject was able to begin their reach with the right hand (see Fig. 2). Subjects were instructed to proceed through the experiment as quickly as possible; however, there was no time limit for tapping the aim location or beginning the reaching movement.

If a subject attempted to begin moving their right hand before an aim location was registered, the message “remember to report aim” was displayed and the trial restarted. While reaching, subjects were instructed to move as quickly as possible past their intended aim location and back to the center in one smooth movement. This “out-and-back” motion was encouraged to decrease the time necessary to return to the center of the workspace. After a successful movement beyond 7 cm, participants were guided back to the start position by a white ring. The ring was centered on the start position and its radius represented the distance of the subjects’ hand to the start position. However, subjects became highly accurate at stopping their out-and-back movement directly on the center of the workspace and used the guiding ring less over time. This greatly increased the number of trials that could be completed in each 1-h session.

During each trial, once the right hand moved out past 7 cm, endpoint feedback was displayed for 500 ms in a position contiguous with the last point of online feedback. If the position of the cursor completely overlapped with the target (<1° of angular deviation), the subject heard a pleasant “ding.” Otherwise, an unpleasant “buzz” sounded. The feedback “too slow” was given if the reach time from start to 7 cm exceeded 600 ms (<1% of trials, these trials were excluded from further analysis).

A familiarization period of 16 trials with online feedback began the experiment. Two baseline periods followed the familiarization period. The first baseline period was composed of 80 trials without feedback to determine whether any subject held strong biomechanical biases that would not be averaged out by the large target set. The second baseline period provided veridical feedback, was also 80 trials long, and was designed to wash out any drift that may have developed in the no-feedback phase. Subjects were not asked to report their aim during these baseline trials as it was assumed that they would always be aiming at the target. A pause was included after the baseline trials so that the experimenter could explain the aiming procedure to the subjects. Subjects were explicitly instructed to indicate their intended reach direction, not the target or the cursor position, by tapping on the aiming ring with their left hand. Following these instructions, subjects completed 16 trials with veridical feedback to familiarize themselves with the touch screen and aiming procedure. The first day concluded with 608 perturbation trials, in which a 45° rotation between movements of the hand and cursor feedback was abruptly introduced. Subjects were provided with a 3-min break at the midpoint of training on each day.

Subjects were carefully observed during the familiarization trials and for the first 50 training trials to ensure that they understood the re-aiming instructions; further direction was provided as necessary during this time. If a subject was observed to “re-aim” repeatedly on the target, instead of adjusting their aim, the subject was stopped and the experimenter ensured that they understood the purpose of the aiming procedure.

On days 2–5, the perturbation was present from the first trial. Subjects were given a quick refresher of the aiming and movement instructions at the beginning of each day and were told that they would “pick up right where they left off” the day before. Subjects completed 800 trials each day, with an ∼3-min break halfway through. A total of 3808 training trials were completed. At the end of day 5, a 32-trial washout period was completed without feedback. During this washout, subjects were instructed to discontinue any strategy they had developed and aim straight for the target.

In experiment 1 (rotation-task), the perturbation was a 45° rotation in either the clockwise or counterclockwise direction (counterbalanced between subjects). In experiment 2, the perturbation was a mirror about the vertical midline. In order to allow comparison between the two experiments, the four targets in experiment 2 (mirror-task) were located 22.5° from the midline so that the solution to the perturbed reach was a 45° angle. The 16 targets in experiment 1 were evenly spaced 22.5° apart on the aiming ring (Fig. 2). Both experiments were programmed such that each of the targets appeared before any were again repeated.

### Data and statistical analyses

The experiment presentation, data collection, and statistical analysis were all completed in MATLAB (The MathWorks, 2016b). During both experiments, the digitizing tablet logged the trajectory of the right hand and the touchscreen monitor recorded the position tapped to indicate the re-aiming location. To allow averaging across targets, hand trajectories were transformed to a common axis with the target at zero degrees. Additionally, the hand trajectories were transformed into heading angles by computing the average angle of the hand from a straight-line path to the target between 1 and 3 cm into movement. This procedure prevents the influence of visual feedback control on estimates of learning. An aiming angle for each trial was defined by the angle between the target and the location tapped on the touchscreen. The implicit process was calculated as the subtraction of the aim angle from the hand heading angle in each trial (Taylor et al., 2014). In all measures, a positive angle represents a counterclockwise divergence from the target. As we measured only two values, and computed the implicit process from those values, we conducted statistical analyses for only the aiming angles and the implicit process angles. We report the mean and standard deviation of hand angles for completeness only. Reaction times were calculated as the interval from target appearance to aim-report, except where noted. All data used in parametric statistical tests were tested for normality using Lilliefors test.

### Modeling

To predict the time courses of explicit and implicit processes in the mirror task, we used a modified version of the two-state model (Smith et al., 2006). Here, we modeled explicit re-aiming as the fast learning process (Xf) and the implicit process as the slow learning process (Xs) over the course of 200 trials (for a detailed account of modeling explicit/implicit learning as fast/slow learning, see previous work by McDougle et al., 2017). In addition, we assumed that explicit re-aiming is updated based on target error, while the implicit process is updated based on the aim-to-cursor distance (Eqs. 1, 2):

(1) Xfn + 1=α f×Xfn + βf×Efn

(2) $$\;Xs\left(n+1\right)=\alpha s\times Xs\left(n\right)+\beta s\times Es\left(n\right).$$

Where Ef is the error between the target and the cursor locations, while Es is the error between explicit re-aiming (Xf)(n) and the cursor location. Note, we did not input actual values of explicit re-aiming in these simulations but instead simply treated them as a faster learning process. In addition, we did not fully collapse the target locations to a common axis to demonstrate how the mirror-reversal perturbation causes different signed errors depending on target location. As such, we separately simulated target locations in the first and third quadrants, and second and fourth quadrants. The values on α f, βf, αs, and βs were determined by hand tuning these parameters to the simple rotation case (rotation-task-rotation-correction model; Figs. 8B, 9*B*; α f=1, βf=0.2, αs=0.98, and βs=0.01). Note, we chose a value for αs that was slightly lower than one to simulate an asymptotic value of the implicit process that is similar to what has been observed experimentally. These same parameter values were then used for both the mirror-task-rotation-correction and the mirror-task-mirror-correction model. In the mirror-task-rotation-correction model, the error for the implicit process was defined in exactly the same way as in the rotation-task-rotation-correction model.

To demonstrate the effect of the implicit process switching the error-correction calculation to be consistent with the mirror reversal, we flipped the sign of the implicit error term in the mirror-task-mirror-correction model (Eq. 3):

(3) Xsn + 1=α s×Xs(n)-βs×Es(n).

The modeling code described here is freely available online at https://osf.io/vtqa3/?view_only=63d8fb50e4574cf986577606707a8e2b. The code is also available as Extended Data 1.

10.1523/ENEURO.0312-20.2021.ed1 Extended Data 1 File Figures_8_9.m contains the modeling code used to generate Figures 8, 9. Download Extended Data 1, ZIP file.

## Results

### Experiment 1

In experiment 1, 12 subjects participated in a visuomotor rotation task for 1 h each day for five consecutive days. Subjects attempted to compensate for a visual perturbation of 45° while reaching to targets that appeared on a circle centered on the start position. To dissociate explicit and implicit learning, subjects reported their intended end position (re-aiming) using a touch-screen monitor (Bond and Taylor, 2017; Hutter and Taylor, 2018; Fig. 2).

**Figure 3.**
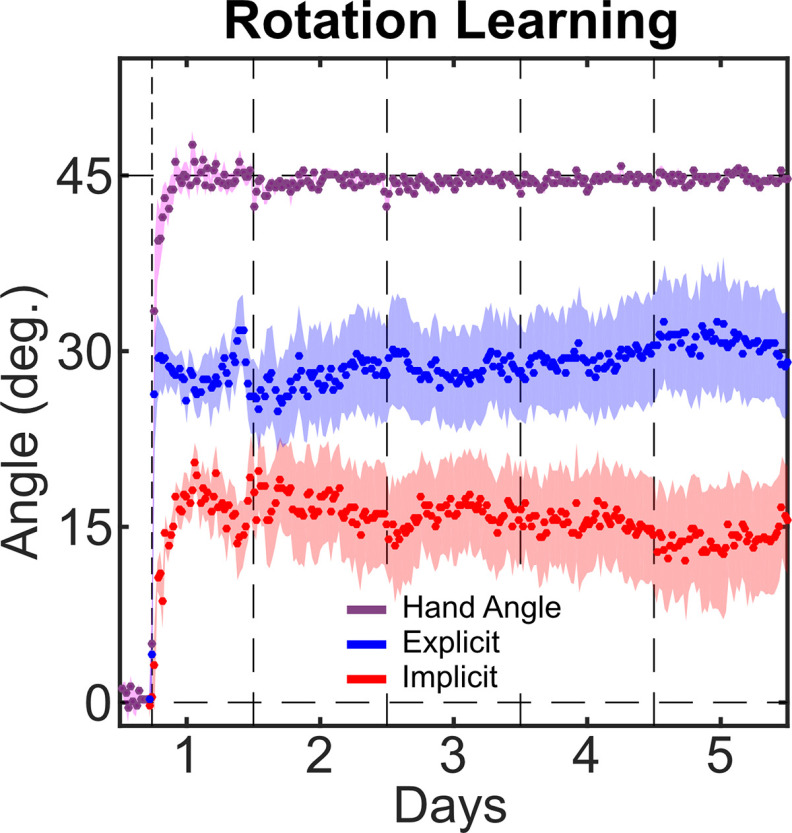
Time course of responses over 5 d of training to a 45° rotation. All responses were rotated to a common axis with the target at 0° and the solution at 45°. The vertical dashed lines represent first the onset of the rotational perturbation, then day breaks. Shaded areas represent between-subject SE.

As expected, participants were able to compensate for the visual perturbation during the first day of training (Fig. 3). Nearly perfect performance, as measured by the hand heading angle, was achieved by the end of the first day (44.8 ± 2.0°) and was maintained in the fifth day of training (44.7 ± 0.8°). Interestingly, this high level of performance was supported by a relative mixing of explicit re-aiming and the implicit process both on the first day (28.1 ± 6.7° re-aiming vs 16.7 ± 6.4° adaptation) and on day 5 of the experiment (30.7 ± 16.2° re-aiming vs 13.9 ± 16.3° adaptation). Note, while it appears that explicit re-aiming accounts for nearly twice as much learning as the implicit process, performing inferential statistics between explicit and implicit learning is theoretically inappropriate since our implicit measure is derived from our explicit measure.

#### Analysis of within-subject and between-subject variance

**Figure 4.**
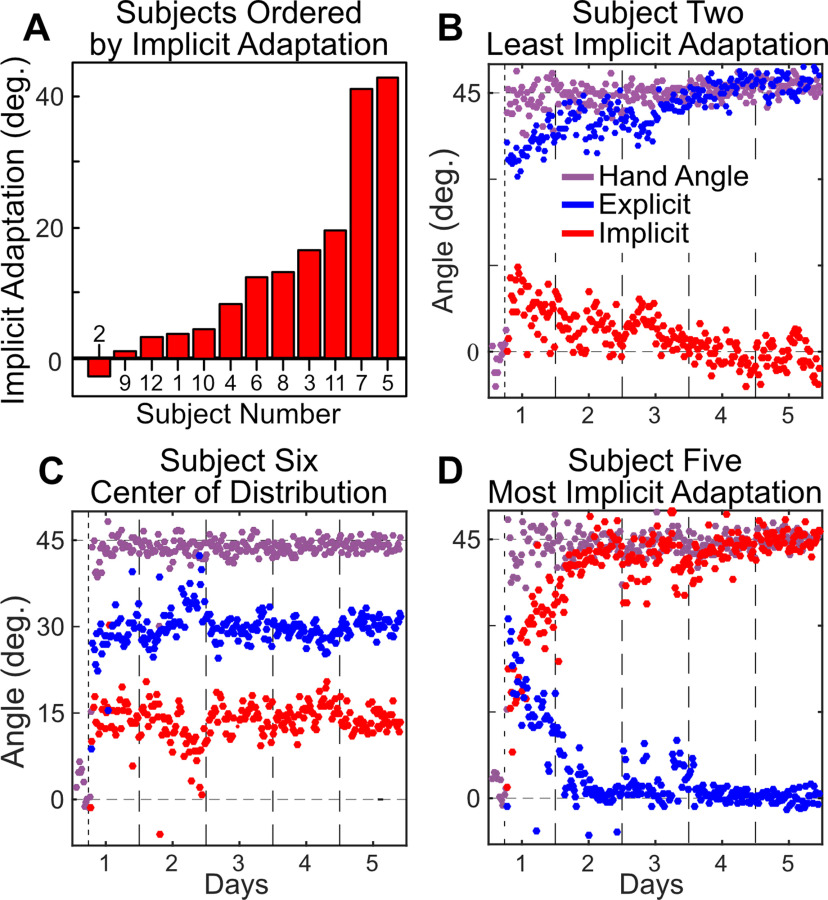
***A***, Bar graph ordering subjects by the average amount of the implicit process as calculated from the last 200 trials of day 5. ***B***, Subject (#2) reporting the most explicit re-aiming at the end of learning, assumed to have the least implicit process learning (i.e., aiming directly at the solution with no perceptible implicit process at the end of day 5). ***C***, Subject (#6) taken from the center of the distribution. ***D***, Subject (#5) reporting the least explicit re-aiming (i.e., aiming at or very near to the target with all learning accounted for by the implicit process).

While learning appears to be divided between explicit and implicit processes, visual inspection shows a notable increase in the between-subject variance for explicit re-aiming and implicit processes following the first day of training. This suggests that the relative contribution of explicit and implicit processes may differ dramatically between subjects. Indeed, if we sort the subjects by amount of the implicit process on day 5, we find a full continuum of learning patterns (Fig. 4). Our 12 subjects run the gamut from no implicit process learning (Fig. 4*B*), through a mixture of the implicit process and explicit re-aiming learning (Fig. 4*C*), to complete implicit process learning (Fig. 4*D*). These results are consistent with the range of the implicit process previously found in numerous studies, including those using standard visuomotor rotation tasks without re-aiming reports (Taylor and Ivry, 2014), perturbations introduced in a pseudorandom walk (Stark-Inbar et al., 2017), and task-irrelevant-visual-error clamp (Morehead et al., 2017; Kim et al., 2018, 2019).

**Figure 5.**
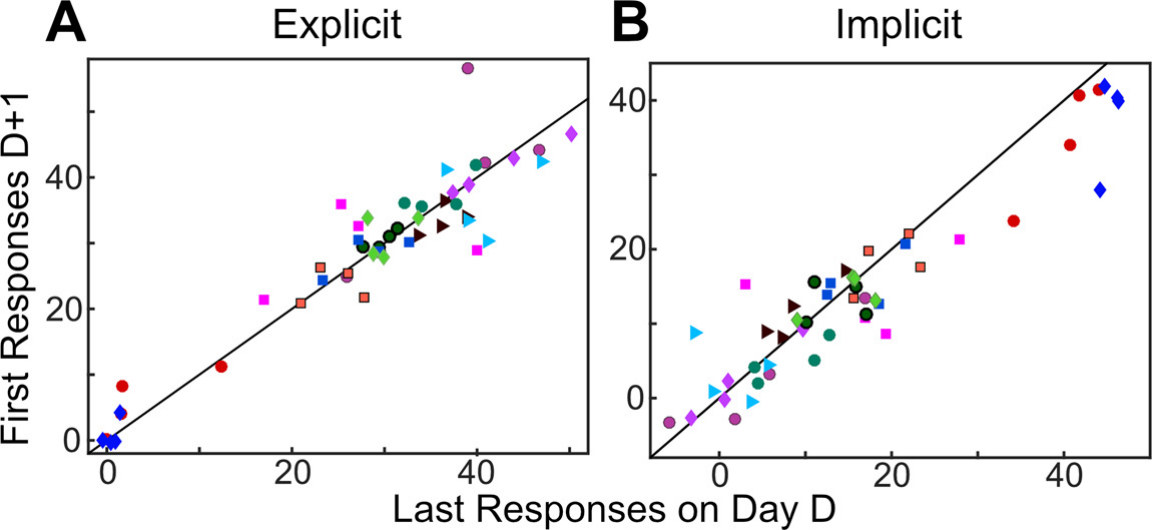
Average explicit re-aiming (***A***) and the implicit process (***B***) in last bin (16 trials) of day D plotted against first bin of day D + 1 (*r *=* *0.94). Each subject contributed four data points and is represented by shape and color for visual clarity. The diagonal lines represent the unity line between responses on D and D + 1. The marker color and shape represent an individual subject across all figures in this manuscript.

Despite these large between-subject differences, participants appeared to be remarkably consistent in maintaining their personal relative contribution of explicit and implicit learning within and across days. To quantify this, we performed a regression comparing explicit re-aiming and the implicit process at the end of each day to the same measure at the beginning of the following day (Fig. 5). We found a high correlation between the end of 1 d and the beginning of the next day for both explicit re-aiming (Pearson’s *r *=* *0.94) and the implicit process (*r *=* *0.94). To underscore that this correlation only reflects consistency at the individual level and not at the group level, we sought to quantify group consistency by shuffling the individual data. Here, we randomly assigned the prior days of each subject to the following days of a different subject 10,000 times. Averaging the correlation over these runs results in a correlation coefficient a fraction of the size seen for the true data (explicit re-aiming average *r* = −0.07; the implicit process average *r* = −0.07).

#### Analysis of adaptation measurements

**Figure 6.**
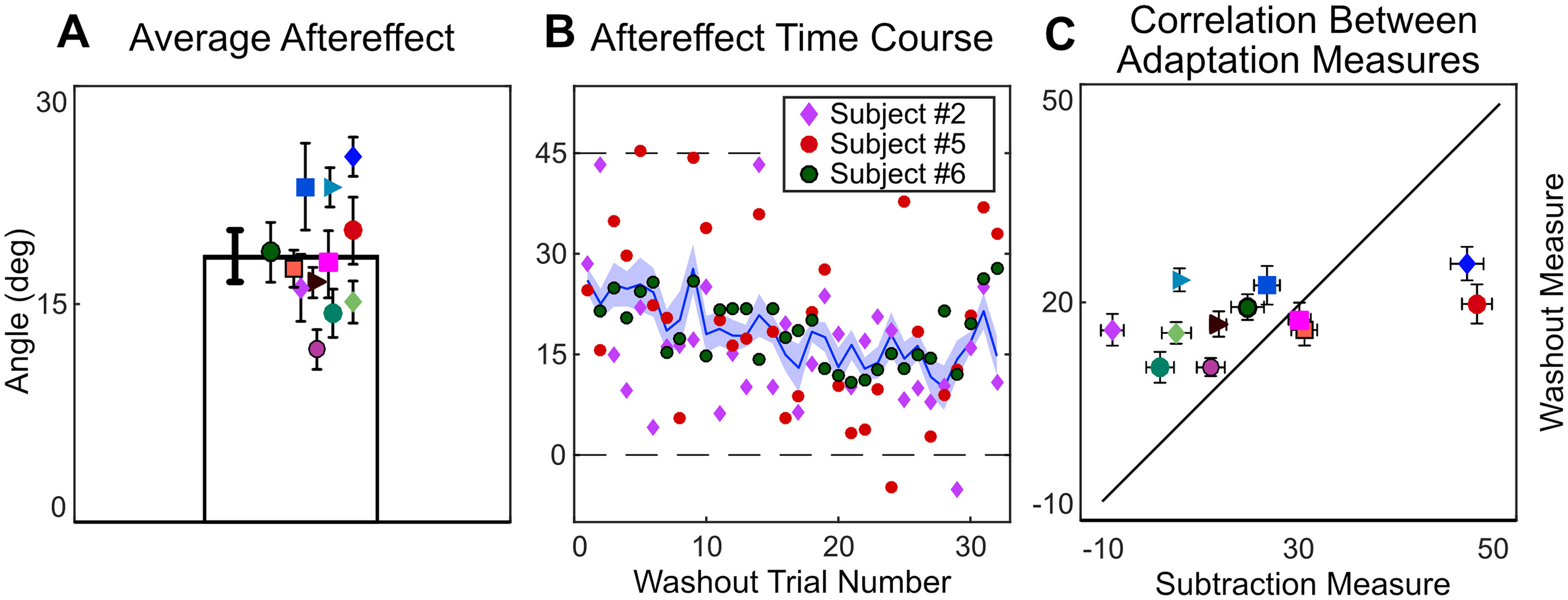
***A***, Average aftereffect for each subject averaged across the 32 trials of no-feedback washout (with subjects instructed to aim and move directly to the target). Error bars represent 1 SEM. ***B***, The time course of washout for the average across subjects, and individual trial values for the three example subjects defined in Figure 6. Shaded area represents between-subjects SE. ***C***, Correlation between each individual’s adaptation as measured via subtraction (hand angle minus explicit re-aiming over the last 32 trials of training) versus washout (average hand angle in 32 washout trials). Error bars represent 1 SEM along each dimension. The marker color and shape represent an individual subject across all figures in this manuscript.

When we use trial-by-trial aiming data to infer the implicit process levels, two subjects appear to compensate fully with the implicit process and five subjects appear to be using a mostly explicit strategy, with the five remaining subjects somewhere in-between. We compared this result with the traditional aftereffects measure, which was completed at the end of the fifth day of training. During washout trials where subjects were instructed to aim directly at the target without using any strategy and on which no feedback was given, not a single subject shows a full 45° aftereffect, or a 0° aftereffect, which would have been indicative of learning entirely via the implicit process and explicit re-aiming strategies respectively (Fig. 6*A*).

It is visually apparent that the aftereffects measure does not display the same range of adaptation that we saw using the aim subtraction method. To quantify this visual observation, we ran a correlation analysis comparing the implicit process in the final 36 trials of training (calculated with the subtraction measure) with the implicit process measured in the first 36 trials of the washout block (calculated by discrepancy from target location, with instruction to reach directly to target). We note that any correlation analyses on a limited sample size (*N* = 12) should be viewed with caution. Additionally, it is important to note that we observed extremely variable trial-to-trial measurements within a subject. While the average adaptation, as reported in most studies of the implicit process, corresponds well to the mean of trial-by-trial adaptation calculated through the aiming subtraction method, the individual measures do not (Fig. 6*B*).

Confidence is warranted in suggesting that some participants fully strategized their way out of the rotation problem. This is because they had to be aware of the correct solution to tell us that they intended to move 45° away from the target. Contrast this with the fully implicit subject, whose explicit reporting behavior (i.e., tapping on or very near the target on each trial) suggests that they were unaware of the rotation. We think that this interpretation is highly unlikely given the size of the rotation. Instead, we think that such behavior is more likely explained by a misinterpretation of the instructions or simple disregard for the instruction. Note, however, participants were required to report their intended aiming strategy on each trial, limiting the effort saved by reporting inaccurately. Additionally, if participants did not understand the instructions, we would expect that they never re-aimed or fully re-aimed, and that this behavior would be consistent from the introduction of the rotation. Instead, we see gradual changes with training. Participants who displayed full implicit process learning followed a consistent time course where the implicit process gradually increased with training while their explicit re-aiming gradually decreased. Likewise, participants who displayed nearly full explicit re-aiming increased their re-aiming angle with training while the implicit process gradually decreased. Importantly, in postparticipation debriefing one of the participants who displayed full implicit process learning indicated that they thought the perturbation was removed; the second such participant declined to be interviewed.

We conducted several *post hoc* analyses in an attempt to determine the cause of the variability in the implicit process found in this experiment. Regression analyses with day 5 of the implicit process regressed by inter-trial interval, reaction time, movement time, or reach end-point variability, all produced null results (*p *>* *0.26). It is likely, however, that this is the result of limited power driven by a small sample size for this type of analysis. A study specifically designed, with much greater power, will be required to identify the source of individual variability.

#### Experiment 1 conclusion

The average response to long-term adaptation in a visuomotor rotation task suggests that both the implicit process and explicit re-aiming continue to play a role in performance even after several days of training. On average, we found the implicit process to be constrained, which is consistent with the findings from studies examining the capacity of single-session implicit learning. However, we saw great variability between subjects, including evidence from two subjects that the implicit process could eventually replace explicit re-aiming.

We next investigate the usefulness of the implicit process in a *de novo* skill learning task, the mirror-reversal task.

### Experiment 2

In experiment 2, 12 subjects participated in a visuomotor mirror-reversal task for five consecutive days. Subjects compensated for a mirror perturbation originating at the midline while reaching to targets that appeared on a circle around the start position. Targets were positioned such that the correct response resulted in a 45° angle between hand and feedback. Subjects reported intended re-aiming using a touch-screen monitor before each trial. The procedure was exactly the same as previously used in experiment 1.

**Figure 7.**
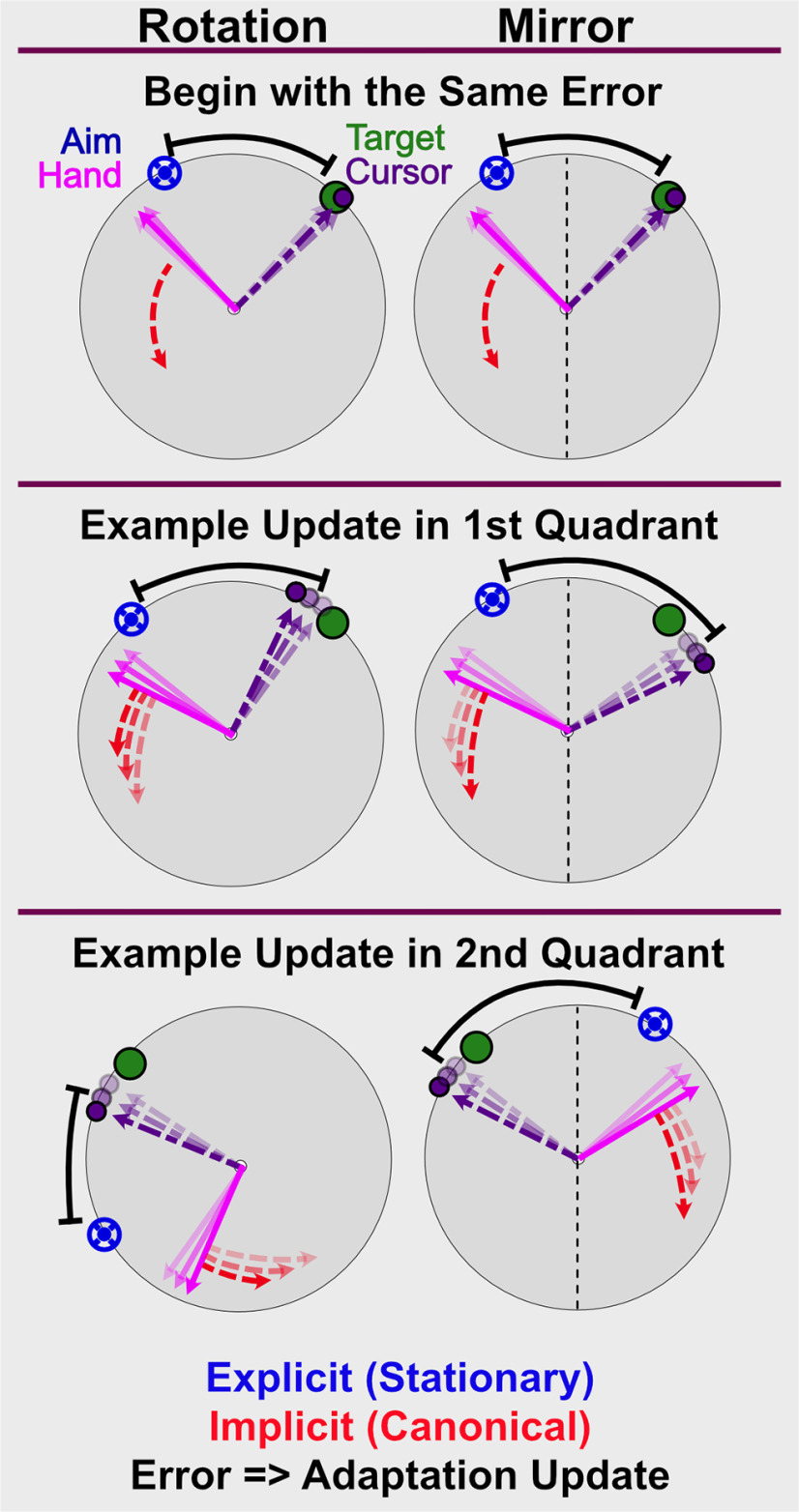
Six scenarios with hand angle (magenta) responding only to the implicit process (red). Explicit strategy (blue) is held constant to simplify the figure. The implicit process (red) is driven by the error (black) between the re-aimed location (blue) and feedback (purple). Additive adaptation pulls the hand away from the re-aimed location and results in decreased error for a rotational perturbation, and increased error under a mirror reversal. The error sign is flipped in quadrants 2 and 4 under a mirror reversal, but not a rotation. Greater transparency of the vectors and endpoints indicates trials further into the past.

As shown in Figure 1, useful implicit learning in rotation paradigms has been described as responding to the discrepancy between the cursor feedback and aiming location (Taylor and Ivry, 2011; Day et al., 2016). For the implicit process to be useful in the mirror-reversal task, the implicit error signal must be calculated in the opposite direction as it is for rotational perturbations (Fig. 7). Additionally, the mirror reversal imposes a counterclockwise error in quadrants 1 and 3, but a clockwise error in quadrants 2 and 4, similar to a dual adaptation paradigm (Howard et al., 2012; Schween et al., 2019). This complicates the transformation space over targets and contributes to the learning difficulty.

For clarity of visualization on graphics, and to underscore the difficulty of accumulating the implicit process during a mirror task, we combined responses for the targets in quadrants 1 and 3 (i.e., at 67.5° with those at 247.5°) and responses for the targets in quadrants 2 and 4 (i.e., at 112.5° with those at 292.5°; Fig. 7). Unlike in the rotation task, normalizing all target locations to zero does not assist in visualizing the data. Statistical analyses were conducted on the full data set, combining all target locations by flipping the sign of quadrants 2 and 4 before averaging.

**Figure 8.**
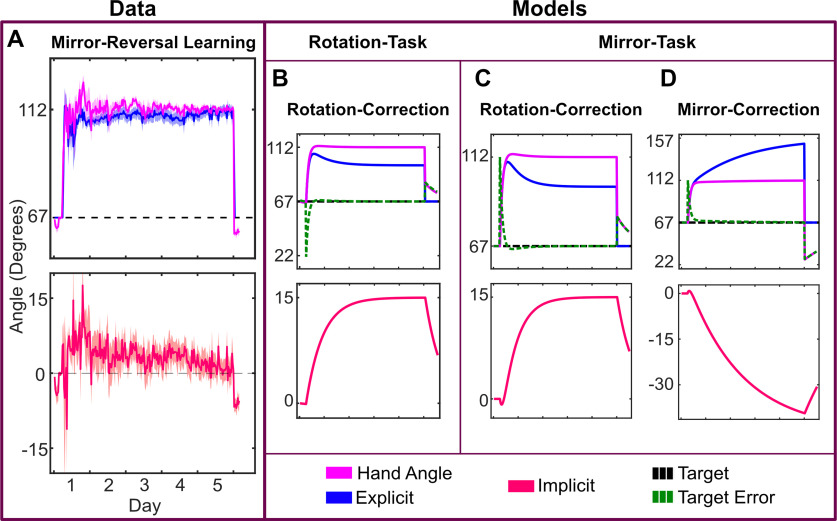
***A***, Mirror reversal learning for all 12 subjects to targets in the first and third quadrants, normalized to 67.5°. The solution is 112.5°. The implicit process is shown separately with positive values being appropriate for a rotational perturbation. ***B***, Rotation-task-rotation-correction model for target at 67.5° with parameters tuned to limit the implicit process to ∼15°. ***C***, Mirror-task-rotation-correction model with parameters defined as in ***B***. ***D***, Mirror-task-mirror-correction model with parameters defined as in previous. Implicit error term is reversed from the rotation correction.

**Figure 9.**
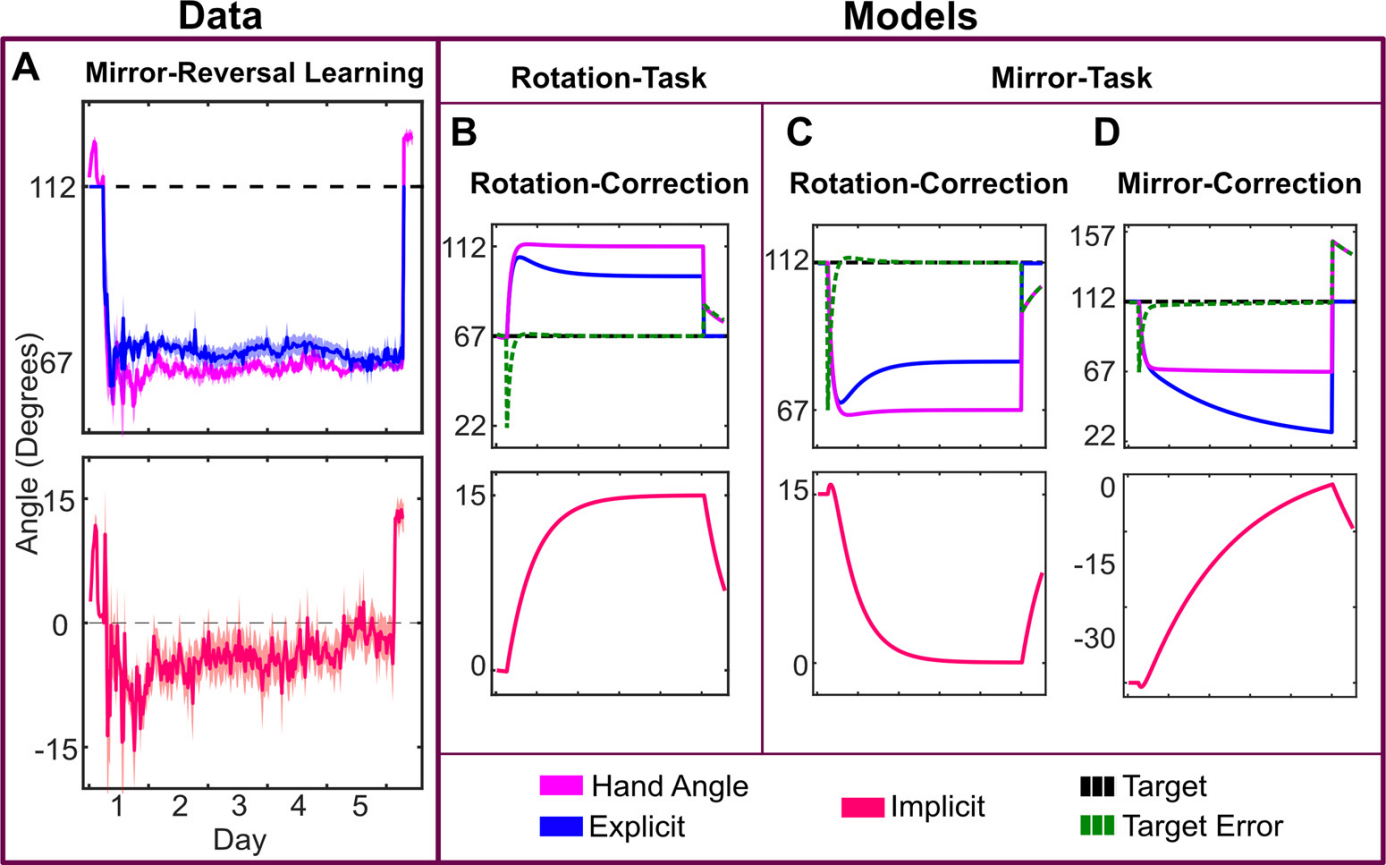
***A***, Mirror reversal learning for all 12 subjects to targets in the second and fourth quadrants, normalized to 112.5°. The solution is 67.5°. The implicit process is shown separately with negative values being consistent with a rotational perturbation. ***B***, Rotation-task-rotation-correction model for target at 112.5° with parameters tuned to limit the implicit process to ∼15°. ***C***, Mirror-task-rotation-correction model with parameters defined as in ***B***. ***D***, Mirror-task-mirror-correction model with parameters defined as in previous. Implicit error term is reversed from the rotation correction.

Unlike the visuomotor rotation in experiment 1, participants on average slightly over-compensated for the mirror-reversal perturbation on the first day (48.5 ± 3.8°); however, performance on the final day was nearly perfect (45.7 ± 1.3°, Figs. 8*A*, 9*A*).

On the first day, performance was again a mixture of the implicit process (8.0 ± 4.6°) and explicit re-aiming (40.5 ± 5.4°). However, while the implicit process initially contributed to learning (*t* test of first day, *t *=* *6.050, *p *<* *0.001), it gradually decreased over time (paired *t* test between adaptation on first and last day, *t *=* *2.202, *p *=* *0.049) and was not significantly different from zero on the final day (2.2 ± 7.8°, *t *=* *0.961, *p *=* *0.357). On average, participants fully compensated for the mirror reversal with explicit re-aiming (43.6 ± 7.6°) on the final day of training. Assuming a constant rate of change and that the implicit process is capped at ∼15° (as seen in experiment 1), the implicit process would become more appropriate (i.e., switch signs) for a mirror reversal after 10–14 d.

#### Analysis of within-subject and between-subject variance

**Figure 10.**
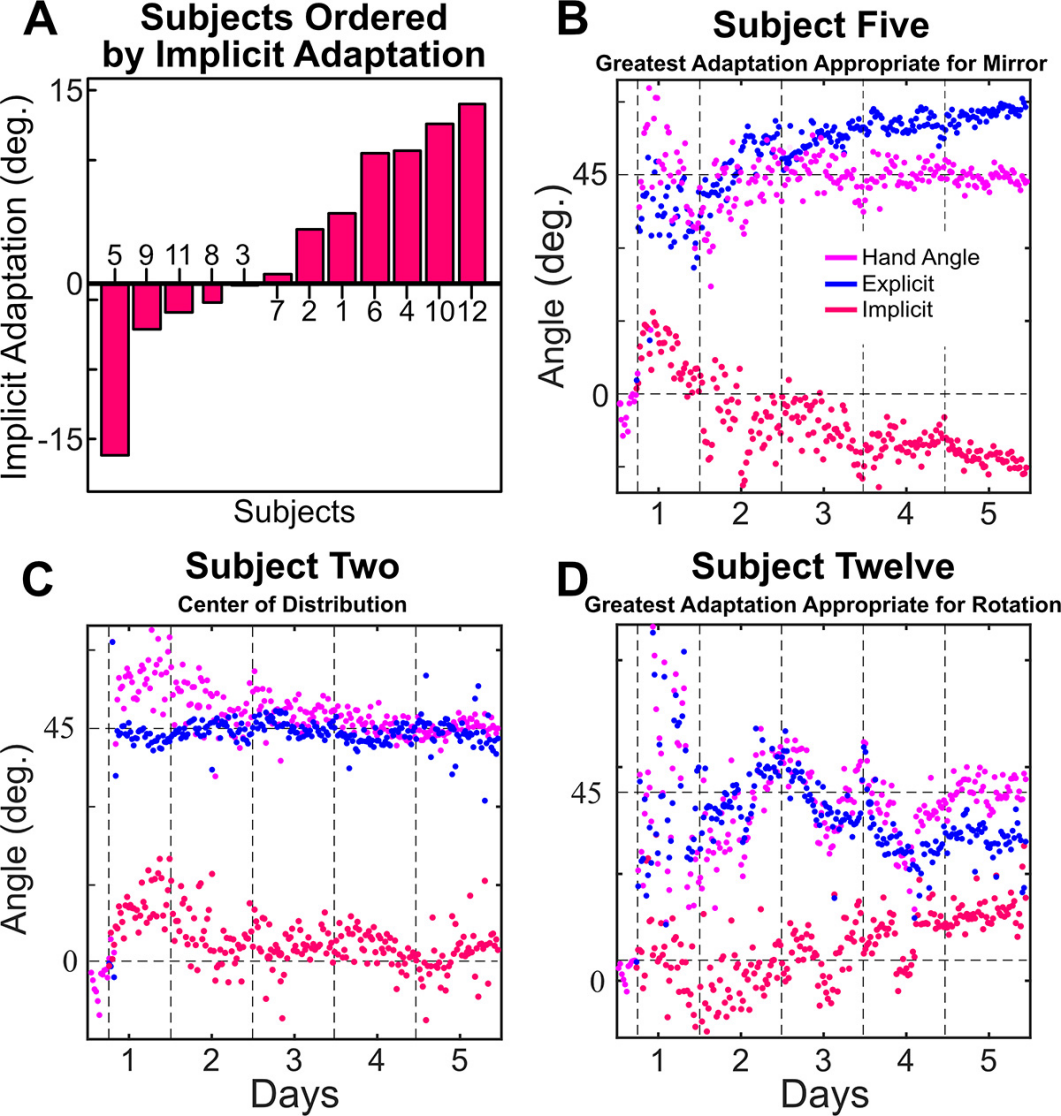
***A***, A bar graph ordering subjects by the average amount of explicit re-aiming that they reported during the last 200 trials of day 5. ***B***, The subject (#5) reporting to have reversed their implicit process so that it is appropriate for the mirror reversal at the end of day 5. ***C***, A subject (#2) representative of the average taken from the middle of the distribution. ***D***, The subject (#12) reporting the most adaptation in the direction that is more appropriate for a rotation.

As with the visuomotor rotation, there was significant variance in how subjects responded to the visuomotor mirror reversal (approximately −15° to 15°; Fig. 10). Subjects were successful through a combination of the implicit process and explicit re-aiming, lost all the implicit process by the end of training, or reversed the direction of the implicit process. Reversed implicit adaptation, visualized as negative, would be appropriate for the mirror-reversal task.

**Figure 11.**
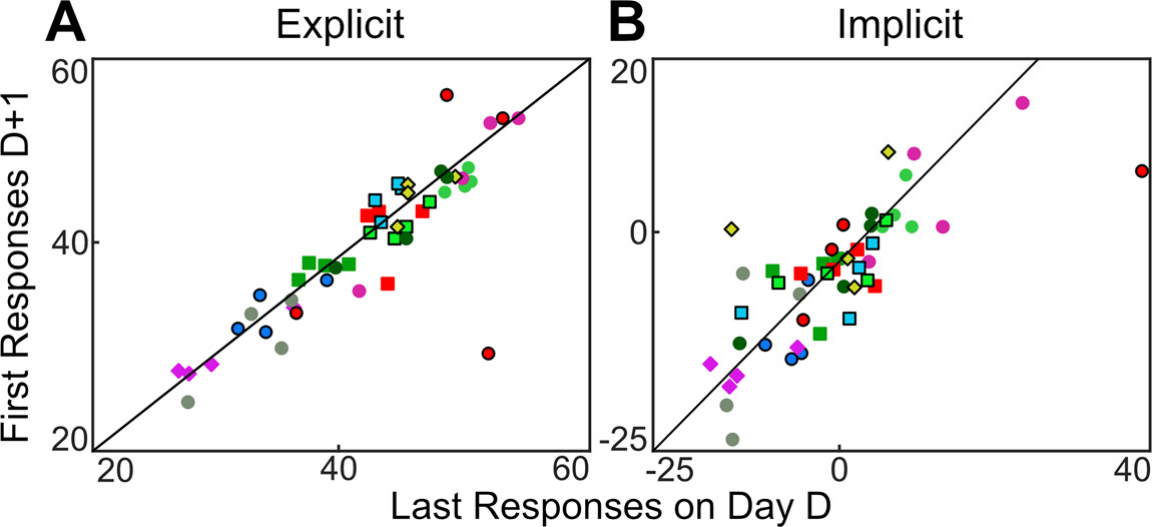
Average explicit re-aiming (***A***) and the implicit process (***B***) in last bin (16 trials) of day D plotted against first bin of day D + 1. Each subject contributed four data points and is represented by shape and color for visual clarity. The diagonal lines represent the unity line between responses on D and D + 1.

As in experiment 1, we conducted a linear regression analysis to determine how consistent subjects were across days (Fig. 11). We again found remarkable consistency within each subject; expressed as a high correlation between the end of 1 d and the beginning of the next day for both explicit re-aiming (*r *=* *0.865) and the implicit process (*r *=* *0.770). When this result was contrasted with a randomization between days D and D + 1, the result is a far worse average correlation than the true data for both explicit re-aiming (average *r* = −0.067) and the implicit process (average *r* = −0.071). Again, while we note that correlations on small sample sizes should be viewed with caution, because of the extremely high correlational relationships we do not think there is reason to believe that the inclusion of more participants would change the conclusion in this case.

#### Analysis of adaptation measurements

Mirror learning has been viewed as a skill learning process, as opposed to adaptation, because it does not result in the same response properties as a visuomotor rotation, such as rate of learning, offline consolidation, and shifts in a speed-accuracy trade-off (Telgen et al., 2014). Given the complexity of forming an intuition about behavior of the implicit process in the mirror-reversal task (Fig. 7), we simulated the potential results using a modified two-state-space model (Smith et al., 2006). The goal was to form graphical representations of responses based on mirror-appropriate adaptation or adaptation to an alternating rotation. The two-state model characterizes learning in visuomotor rotation tasks in terms of two separate learning processes: one that learns slowly but retains memory and the other learns fast but quickly forgets. Recent work suggests that the slow process might be the implicit process while the fast process is explicit re-aiming (McDougle et al., 2015). We adopted the conventions of this model to predict learning based on the sign of the error signal. Note, our goal here is not to model mirror-reversal learning per se, but to determine whether the pattern of the implicit process we observed is more consistent with learning appropriately for a mirror reversal or for a visuomotor rotation.

Within the model, we assumed that the explicit re-aiming is updated based on target error, while the implicit process is updated based on the angle between the aim and the cursor (for details, see Materials and Methods). The learning and forgetting rates were determined by tuning these parameters to the simple rotation case to cap the implicit process at ∼15° (rotation-task-rotation-correction model; Figs. 8*B*, 9*B*; Morehead and Smith, 2017). These same parameter values were then used for both of the mirror models. In the mirror-task-rotation-correction model, the error for the implicit process was defined in exactly the same way as in the rotation-task-rotation-correction model (Figs. 8*C*, 9*C*). However, to demonstrate the effect of the implicit process switching the error-correction calculation to be consistent with the mirror reversal, we flipped the sign of the implicit error term in the mirror-task-mirror-correction model (Figs. 8*D*, 9*D*).

When the mirror task is simulated with error calculated as appropriate for a rotation [aim-cursor], the result is a response pattern much like that of the data. In contrast, an error calculation that is appropriate for the mirror [-(aim-cursor)], produces results in a radically different pattern from the data (compare Figs. 8*A–D* and 9*A–D*). The modeling results demonstrate that the implicit process under a mirror reversal first operates as if the perturbation was rotational in nature, then suppresses this adaptation.

**Figure 12.**
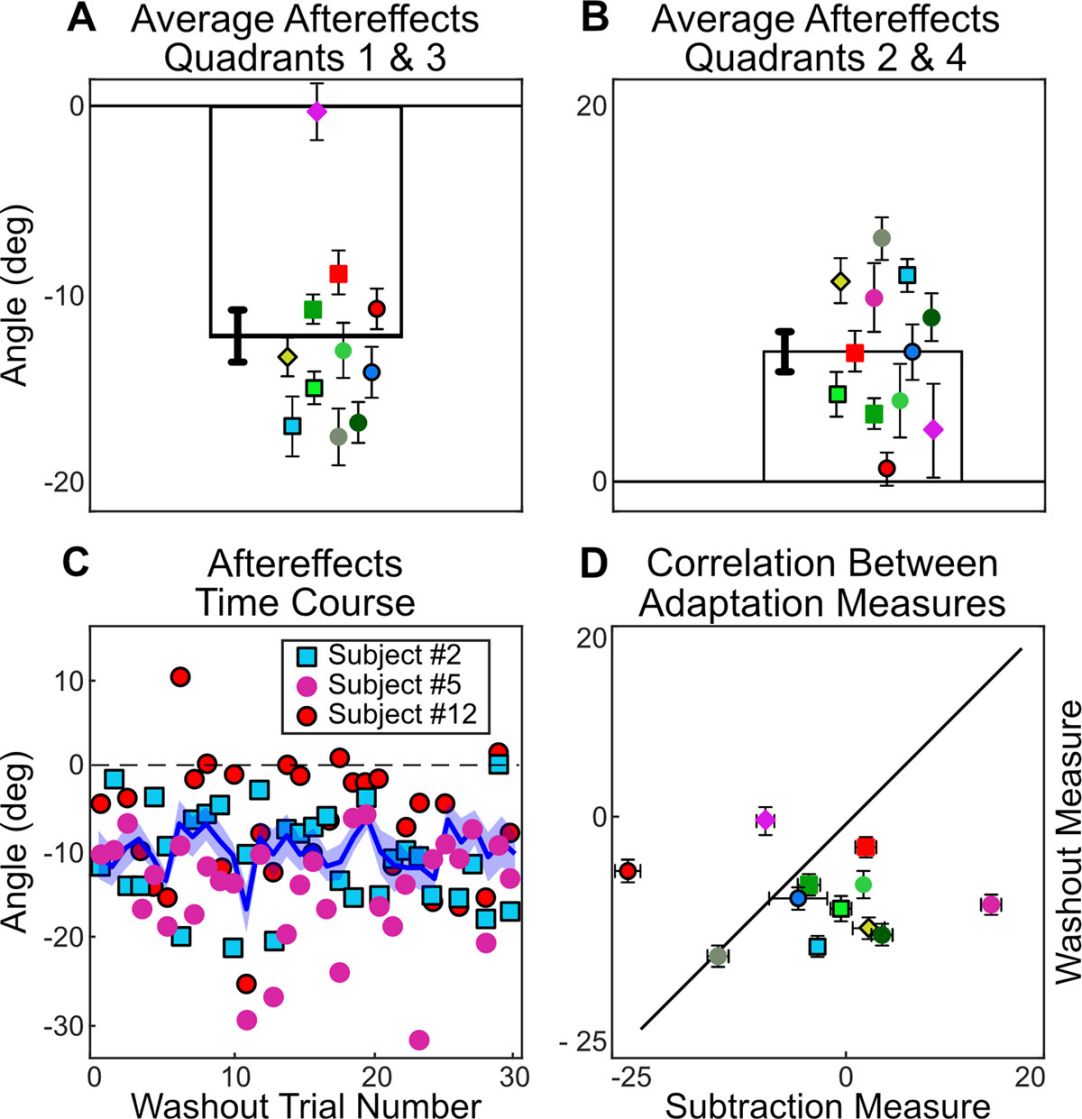
Average corrected aftereffects for each subject across the 32 trials of no-feedback, washout (with subjects instructed to aim and move directly to the target) in (***A***) quadrants 1 and 3 and (***B***) quadrants 2 and 4. Each subject is jittered left to right in order of least to most explicit re-aiming at the end of day 5. ***C***, The time course of washout for the average across subjects and individual trial values for three representative subjects [#5 (lowest), #2 (median), #12 (highest), re-aiming on last day]. The shaded area represents the SEM for all subjects. ***D***, Correlation between the implicit process revealed by the subtraction method during training and aftereffects during the washout phase.

In the washout phase, at the end of day 5, subjects were instructed to aim directly at the target without using any strategy to measure the size of the aftereffect. Similar to experiment 1, we observed a very limited range of aftereffects compared with the implicit process calculated throughout training. After correcting for baseline bias, aftereffects hover slightly, yet significantly, below zero (average corrected aftereffect = −3.06 ± 3.2°, *t* value = −3.32, *p* = 0.007; Fig. 12). As in experiment 1, subjects were instructed to aim directly at the target without using any strategy in washout trials and no feedback was given. In addition to exhibiting the high within-subject variance seen in experiment 1, the washout phase was confounded by the discrepancy between aiming to hit a target in training versus aiming to hit that same target in washout. Adaptation appears to be centered around the aim location, not the target location (Day et al., 2016; McDougle and Taylor, 2019; Schween et al., 2019). In our task, a correct re-aiming location in training is approximately the same location as another target on the opposite side of the mirror axis. The new aiming location in washout (i.e., directly at the target) is at the same location as that for the opposite target in training. Adaptation that resulted in positive aftereffects (relative to the adaptation expected in a visuomotor rotation task) during training would have resulted in negative aftereffects in washout. This suggests that we should read the average aftereffect as being in the direction to correct for a rotational error, not a mirror reversal.

### Comparison of the results of experiments 1 and 2

Although experiments 1 and 2 were conducted sequentially, and therefore should not be statistically compared, we took care to keep both experiments as similar as possible to allow qualitative comparison.

**Figure 13.**
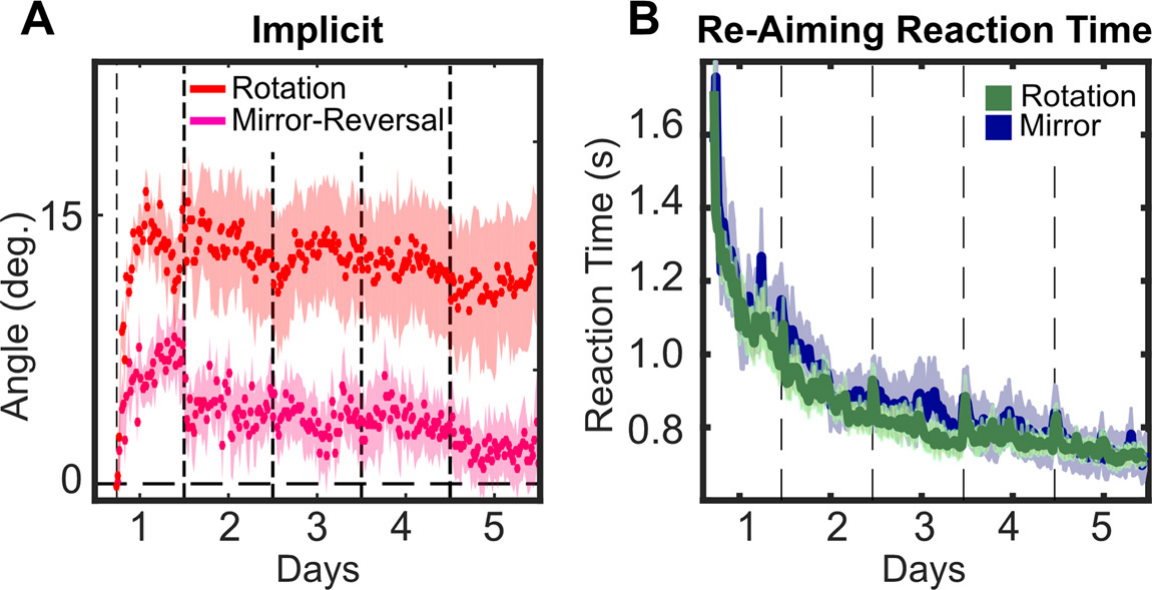
Comparison between subjects in experiment 1 (rotation) and experiment 2 (mirror reversal). The vertical dashed lines represent first the onset of the perturbation, then day break. The shaded areas represent the SEM. ***A***, Time course of the implicit process measured via subtraction from explicit re-aiming during training. ***B***, Reaction time across training calculated as interval from target onset to re-aim registered. Because re-aiming was introduced right before perturbation onset, the time course starts when the perturbation was initiated.

We saw a steadily declining (average) implicit process for subjects training to a mirror reversal in experiment 2. This is in contrast to the flat average of the implicit process function seen for the duration of training to a visuomotor rotation. A visual comparison of these time courses shows starkly different behavior between the two groups (Fig. 13*A*). While subjects completing the mirror-reversal task did not, on average, reverse their implicit process sufficiently for it to work with their learning goal, they did suppress adaptation when compared with the subjects learning a rotation. This suppression is evident from the first day of training, where we see a slowing of initial adaptation rate. The source of this suppression remains an open question (see Discussion).

We find that re-aiming reaction times, the time between target appearance and the touchscreen registering a re-aim tap, gradually decrease to approximately half over the course of 5 d of training (1.4 ± 0.2 s in first five bins of rotation training to 0.7 ± 0.07 s in last five bins of rotation training; Fig. 13*B*) with no difference between the groups.

## Discussion

The idea that adaptation of the motor system to altered feedback is largely the result of the implicit process can be dated as far back as Stratton (1896). Our everyday experiences reflect this view. For example, compensating for the lateral and vertical shift of a computer mouse or a new pair of eyeglasses does not feel like the result of explicit processes. However, previous studies that have sought to isolate implicit learning in visuomotor adaptation tasks have found that it appears to be limited (Bond and Taylor, 2015; Morehead et al., 2017; Kim et al., 2018; Krakauer et al., 2019). Here, we sought to determine whether the limited capacity of the implicit process could be overcome with extended training. In two experiments, we find that learning of the implicit process to both a visuomotor rotation and mirror reversal remains limited, for the majority of participants, after 5 d of training.

### Limitation of the implicit process and measurement reliability

In these experiments, we measured the implicit process by subtraction from explicitly indicated re-aiming behavior and as an aftereffect (Taylor et al., 2014; Bond and Taylor, 2017). Regardless of the metric used to measure the implicit process, we found that implicit processes do not fully account for learning in a rotation task. The implicit process in the mirror reversal task was initially counterproductive to performance before becoming suppressed with increased training. These findings may have been expected given the recent evidence that the implicit process falls well short of full learning in visuomotor adaptation tasks (Morehead and Smith, 2017; Morehead et al., 2017; Kim et al., 2018, 2019) and in the wrong direction in the mirror reversal task (Gritsenko and Kalaska, 2010; Lillicrap et al., 2013; Telgen et al., 2014; Kasuga et al., 2015; Hadjiosif et al., 2021). Nonetheless, we thought that this limitation of implicit process may be overcome with more training, an idea that failed to be supported by our 5-d experiments.

While group-level data do not provide evidence that the implicit process is sufficient for complete learning in a visuomotor rotation task, certain individuals do show complete adaptation under the subtraction measure. Enormous variability, spanning the entire range from zero to full adaptation through the implicit process, was seen across individuals. This suggests that the traditional method of averaging over participants is simplifying, and perhaps obscuring, experimental results (Gallistel et al., 2004). It remains an open question as to why there is such a degree of inter-subject variability; however, this degree of variability has been observed in several studies where even for small rotations, on the order of 3–10°, they found implicit learning ranging from nearly zero to almost 50° (Stark-Inbar et al., 2017; Morehead et al., 2017; Kim et al., 2018, 2019). Tsay et al. (2021) found a modest yet significant correlation between the degree of proprioceptive variability and realignment with the degree of implicit learning. This finding is consistent with Bayesian sensorimotor integration framework where adaptation is a function of the weighting between proprioception and vision based on their relative degrees of noise. Participants with more noise in their proprioceptive system may be more strongly driven by visual feedback, resulting in greater implicit learning for visuomotor perturbation.

An additional possibility for the limited degree of implicit learning observed here, as well as the variability between participants, may be the result of explicit strategy use. While several studies suggested a considerable degree of independence between implicit and explicit processes (Mazzoni and Krakauer, 2006; Taylor and Ivry, 2011; Taylor et al., 2014), this has recently been challenged. Participants who reported their explicit aiming direction throughout training, showed less implicit learning than participants who were only probed at the end of training (Maresch et al., 2021). By asking participants to report their aiming location on each trial, learning may be shifted more toward explicit strategies. It should be noted that in the original study that developed the aim report method, there was little difference in the size of aftereffects between groups that reported on each trial versus never reported (Taylor et al., 2014). This was, in part, our reasoning for not including a no-report control group in conjunction with the difficulty of recruiting participants for a 5-d study where the primary dependent measure (i.e., aftereffects) occurs only once at the end of the study. What is more, like Maresch et al. (2021), we too find no correlation between the degree of implicit adaptation measured via a subtraction measure during training and the size of the aftereffect. Nonetheless, implicit adaptation appeared limited in both measures. Clearly, more work is needed to flesh out this psychological equivalent of the measurement problem.

Finally, if the implicit process and explicit aiming operate on the same target error signal, then learning by one system would compete with the other (Albert et al., 2021). Indeed, there appears to be a reliable negative correlation between learning by explicit re-aiming and the implicit process (Albert et al., 2021); however, this is difficult to interpret since both processes are linked to cursor feedback and if the implicit process has plateaued, then more explicit re-aiming would be required to restore performance. What is more, the initial findings by Mazzoni and Krakauer (2006) strongly suggested that the implicit process operates on a sensory-prediction error and not target error, but this too has recently been challenged (Ranjan and Smith, 2020; Albert et al., 2021).

### The implicit process in the mirror-reversal task

The mirror-reversal results present an exciting future opportunity to determine the error signal that initiates suppression of the implicit process in the mirror-reversal task. Large variability in individual behavior provides a foothold to understanding suppression of implicit processes. Here, we consider a few possible explanations that could be a target for future research.

The mirror reversal, unlike a rotation task, has an error-signal with a constantly switching sign. Previous work has shown that inconsistency in error signals reduces overall learning (Castro et al., 2014) and the implicit process in particular (Hutter and Taylor, 2018), which is consistent with the motor system storing a history of errors (Herzfeld et al., 2014). Here, we would expect participants that experienced more error switching, through their unique history of missing the target, to suppress implicit adaptation more quickly and fully (Albert et al., 2021).

Second, the difference between rotations and mirror reversals could be thought of as a reparameterization, as previously considered in rotations, compared with learning a completely new structure, suggested for the mirror reversal (Braun et al., 2009). The reparameterization of the transformation matrix, as seen in adaptation to a rotation, might be the fundamental function of the implicit process. Indeed, the implicit process may be exceptional at matrix reparameterization. In contrast, learning a mirror reversal requires learning the full structure of the task, including the relationship between parameters in the transformation matrix. It appears that the implicit process tries to reparametrize the transformation matrix as if it were a rotation even if doing so is inappropriate for the task. Only after days of training does the implicit process appear to stop reparametrizing inappropriately. It could be that once a new structure is specified, the structure of the mirror reversal, the implicit process becomes appropriate for this new structure. The speed of learning this structure would dictate how quickly implicit processes began reparametrizing appropriately.

A slightly lower-level description of this problem, one rooted in control engineering, was put forth in a recent study by Hadjiosif et al. (2021), which compared adaptation under a visuomotor rotation and a mirror reversal as a way to distinguish between updating a forward model and updating a control policy. They too find that the implicit process in a mirror reversal was directed in the wrong direction to counter a mirror reversal, which is inconsistent with the updating a forward model. Instead, implicit learning of a mirror reversal may require direct updating of a control policy. Learning a new control policy would again have the outcome of changing the structure of the transformation matrix.

These explanations are highly speculative, and additional research is necessary to pin down the reason for the implicit process being counterproductive in a mirror-reversal task, and the subsequent suppression of the implicit process.

### Proceduralization of explicit re-aiming

Although the implicit process is insufficient for learning, we suspect that it is highly unlikely that subjects are performing a time-intensive, computationally-demanding, strategizing process at the end of 5 d of training. A more likely explanation is that the re-aiming strategy has become partially proceduralized, also referred to as caching or habitualization (Huberdeau et al., 2019). We would expect to find evidence for proceduralization in participants’ reaction or preparation times (Logan, 1980; Cohen et al., 1990). Reaction time increases are commonly observed at the onset of visuomotor perturbations and gently decline with training (Saijo and Gomi, 2010; Fernandez-Ruiz et al., 2011; McDougle and Taylor, 2019). If preparation time is limited, performance is significantly hindered in visuomotor rotation tasks as participants may not have sufficient time to re-aim their movements to counter the rotation (Fernandez-Ruiz et al., 2011; Haith et al., 2015). However, one study found that performance under constrained preparation time can be restored following 2 d of training, potentially reflecting a proceduralization of the re-aiming strategy (Huberdeau et al., 2019).

Here, we find that re-aiming reaction times in both experiments gradually decrease to approximately half over the course of 5 d of training (1.4 ± 0.2 s in first five bins of rotation training to 0.7 ± 0.07 s in last five bins of rotation training; Fig. 13*B*). We would like to interpret this result as indicative of proceduralization of the re-aiming strategy in both tasks. Note, the absolute value of reaction time during training is expected to differ from previous reports, as our measure of reaction time necessarily includes the time taken to report the re-aiming location with the non-dominate hand (Fernandez-Ruiz et al., 2011; Haith et al., 2015). Nonetheless, the fact that we observe a significant drop in reaction times over the course of training, and given the traditional view of interpreting reaction times as reflective of computational demands, we suspect that this is an indication that proceduralization has occurred.

The question turns to what might underlie proceduralization of explicit re-aiming. One possibility is that the process is as simple as forming a stimulus-response mapping, allowing subjects to automatically perform an action that was previously conducted through computation (Daw et al., 2005). Additionally, stimulus-response mappings may result in a cached response or habit, which could simulate an implicit process by being extremely fast and relatively robust to added cognitive loads (Daw et al., 2005; Dayan, 2009; Haith and Krakauer, 2013). McDougle and Taylor (2019) found evidence that initial rotation re-aiming strategies eventually give way to a theorized look-up table for each target location (a stimulus-response map), echoing Logan’s dual process theory of skill as reflecting a shift from algorithmic-based to retrieval-based operations (Logan, 1988).

In summary, in these two experiments, we found that, on average, the implicit process does not account for the majority of adaptive learning, even after many days of consolidation. However, we also saw large variation between individual subjects. This variation questions the validity of averaging over subjects to make claims about implicit processes, and raises questions as to why one measure captures this variability while the other does not. Finally, we found evidence in the mirror-reversal task that the implicit process is slowly suppressed to compensate for alternate task demands.

## Synthesis

Reviewing Editor: Nicholas J. Priebe, University of Texas at Austin Decisions are customarily a result of the Reviewing Editor and the peer reviewers coming together and discussing their recommendations until a consensus is reached. When revisions are invited, a fact-based synthesis statement explaining their decision and outlining what is needed to prepare a revision will be listed below. The following reviewer(s) agreed to reveal their identity: Opher Donchin, Pierre-Michel Bernier.

SYNTHESIS

The reviewers highlighted the high quality and difficulty of the experiments and recognized the importance the work in understanding the role of implicit adaptation in motor learning. The reviewers noted the particular significance of the reduction in implicit learning for the mirror reversal task. Both reviewers also noted, however, significant issues related to the conclusions that are being drawn from the results. Both reviewers had major comments about the current exposition, though there is some difference in perspective about the degree to which adaptation may contribute to de novo motor learning. Both reviewers were concerned by the large variability across subjects, particularly given the small sample size, and felt that this variation in behavior was not discussed in sufficient detail. The reviewers provide detailed suggestions regarding these and other important issues, which you should find useful in revising your manuscript.

Reviewer 1

ADVANCES THE FIELD

The data collected is difficult to collect because it requires 5 consecutive days of experiments in each subject. In addition, the experiments seem performed well and data quality seems high.

STATISTICS

A power analysis is required (as detailed in comments to authors) and some error bars are missing in the plots.

SOFTWARE

We downloaded and tried to run Figures_8_9.m but received an error “Unrecognized function or variable ‘plot_format’.”

COMMENTS

The manuscript explores how practice over several consecutive days impacts implicit adaptation in two different tasks: a visuomotor rotation task and a mirror task. In the rotation task, implicit adaptation plateaus after the first day of training and afterwards remains constant. In the mirror task, implicit adaptation drifts towards 0 throughout the entire practice. The central claim of the paper is that in neither task does 5 days of training lead to a marked increase in the amount of implicit adaptation. For visuomotor rotation, the failure of the implicit adaptation to increase is interpreted to mean that implicit adaptation is limited in its ability to reach full adaptation. In the mirror reversal task, the authors interpret their result to mean that implicit adaptation has no ability to successfully adapt to mirror reversal. Taken together, the results lead the authors to question the utility of implicit adaptation for the broader spectrum of motor skill learning.

The manuscript targets a key area of exploration in the study of motor control. The authors are correct that extrapolating findings about implicit adaptation from visuomotor rotation to other motor tasks, such as the mirror task, can provide valuable insights into the mechanisms of implicit adaptation in general motor learning. However, we have reservations about the work as it currently stands. These can be broadly categorized into issues with the experimental design, the statistics, the interpretation of the existing literature and the interpretation of the results. We detail the major and minor points below.

Major issues

Existing literature:

In discussing the evidence that adaptation only plays a minor role in visuomotor rotations (lines 38-39) the authors only provide support for a weaker claim: that explicit re-aiming does play a role. Indeed, the Mazzoni and Krakauer (2006) paper they cite shows implicit adaptation can override explicit strategies. The authors should also relate to recent evidence showing that long-term retention (in the form of savings) can actually proceed without formation or recalling of explicit strategies (Yin et al. 2020). We feel (as discussed below) that the authors need to clarify the point they feel is made by the experiments and then reassess how their experiments will extend the existing literature. Two additional papers should also be discussed:

Telgen J Neurosci 2014 (https://www.jneurosci.org/content/34/41/13768)

Yang bioRxiv 2020 (https://www.biorxiv.org/content/10.1101/2020.01.15.906545v1.full)

Experimental design:

The authors set out their question in the introduction: can implicit adaptation can lead to long term motor learning in the absence of explicit re-aiming. The experimental design does not seem to answer this question. There is simply no condition with an absence of explicit re-aiming. When this question has been asked (see Yin 2020 referenced above), it has been answered in the affirmative. Again, the authors should clarify which question their data addresses and make a strong link between the question and the experimental design.

Indeed, the experimental design seems to encourage explicit re-aiming. For example, it has been shown previously that reporting can affect different aspects of adaptation: it promotes full asymptote (see preprint by Langsdorf et al. 2020), it has been associated with more explicit re-aiming and less implicit adaptation (see Leow et al. 2018 and preprint by Maresch et al. 2020). Moreover, large rotations (Bond et al. 2015) that are introduced abruptly (Werner et al. 2015) also cause more explicit re-aiming. This suggests that possibility that the authors consider collecting data in conditions which are known to contribute to stronger implicit learning. At the least, this should be discussed as a limitation of this study.

Along similar lines, the authors argue (line 44) that because implicit adaptation is only partial, it cannot lead to long-term motor learning. This seems like a strange claim. Can it be justified?

The claim that the authors can make on the basis of this design is that not all explicit learning eventually becomes implicit. This seems like a straw man argument. Even if it is taken seriously, it may be that more than 5 days are necessary to make some tasks fully implicit. The Stratton (1896) and Kohler (1941) studies which led the authors to choose 5 days actually used five full days of continuous exposure. This is much more exposure than used in the current study. We imagine that extending the experiments to more than 5 days or trying to have subjects practice for entire days will not be practical, but this limitation does need to be discussed.

Statistics:

It would be helpful to present a power analysis that would show that this sample size is large enough to show an effect of at least 2 - 3 degrees. If not, the authors should consider collecting additional data to achieve 90% power for an effect size of 2-3 degrees. In this case, many would argue that the additional subjects should be analyzed separately in order to preserve the purity of the statistical approach. The power of the original study for a small effect size should be reported.

Figures 6 & 10: Error bars on the bar for the mean and individual subject error bars would be helpful. Also, a scatterplot of implicit adaptation vs aftereffect with error bars in both directions would give a better indication of how these measures relate to each other.

Lines 328 - 329: if arguing for similarity, an equivalence test should be conducted (Lakens Advances in Methods and Practices in Psychological Science 2018 has a nice tutorial).

Figure 10: this figure is mean to show that aftereffects are in the direction opposite to the appropriate direction, suggesting an inability of subjects to change the way their implicit system corrects. This argument would be made more powerful if the results were broken down by quadrants, to show that for each the corrections were in the “standard” and not the “reversed” direction.

Discussion:

The discussion, and the paper generally, feels to us that it glosses over the inter-subject differences. Individual subject variability was quite pronounced: some subjects show almost 45{degree sign} of implicit adaptation and others close to 0{degree sign} in the rotation task. The sample size is very small and the data is very noisy (as shown by the large between-subject variance). In line 233, the SEM of re-aiming and implicit adaptation is around 16{degree sign}, meaning implicit adaptation could be as big as explicit re-aiming in some cases. In the mirror task, some subjects show implicit adaptation appropriate for the mirror task and others do not. This is especially important since task details could affect the proportions of subjects showing more implicit adaptation in the two tasks. The discussion should fully address both intersubject variability and the possibility that subtle details of task design may be influencing the results.

The discussion should address the authors expectation (lines 331-334) that implicit adaptation should become more appropriate for the mirror reversal task after more days of training. What are the two alternative theories of how implicit adaptation might work with regards to the mirror task and how do they lead to different expected results. Once this is discussed, the actual results can be understood in the correct context.

Much of the discussion (lines 443-512) is devoted to the topic of proceduralization and theories of motor learning. This seems off topic both in terms of the central question in the manuscript and the design of the experiments. It also seems largely speculative. We recommend reducing this part of the discussion to no more than 20 lines (slightly less than a page).

There is no clearly set out discussion of the studies limitations. This should be added. It should discuss, at the least, the intersubject variability, the relatively small number of subjects, the relatively limited amount of training in the tasks, and the interpretational difficulties associated with measures of explicit and implicit adaptation.

Minor comments

Line 44: asymptotic adaptation remained partial instead of insufficient?

The authors’ continuous implicit measure is solely derived from explicit reports. They also use aftereffects as a measure of the final implicit adaptation. This can be problematic since these measures have been found to differ profoundly in many situations (Taylor et al. 2014, Bond et al. 2015, Leow et al. 2015, Bromberg et al. 2019). The authors themselves find a mismatch between these two measures. It has been suggested that implicit adaptation as derived from reporting may not measure the same as the aftereffect (see preprint by Maresch et al. 2020). This mismatch between measures should be addressed in the discussion of possible limitations of the study.

Many analyses are not described in the methods (individual subject differences, regression comparing adaptation at the end of each day to beginning of next day, comparison between implicit and aftereffect on day 5). These should be included in the methods section.

Was online or endpoint feedback used during rotation trials? The description is somewhat confusing: line 125: a small circular cursor (0.15 cm 125 radius) provided online feedback and then line 136: endpoint feedback was displayed for 500 ms.

Is there a reason that 12 targets were used in Experiment 1? How many targets in Experiment 2 (I think 4, but it’s not explicitly stated)?

Reaction time calculation line 186-187: Reaction times were calculated as the interval from target appearance to aim-report, except where noted. The authors call the “re-aiming reaction time”. While this seems appropriate, I’m still wondering in how far this really reflects an actual reaction time. A short discussion about what this type of reaction time means and how it differs with other reaction time calculations would be desirable.

Line 233: How is the SEM calculated? Did the authors account for within subject variability?

Line 235-237: the authors are correct that a direct comparison of explicit and implicit is inappropriate. However, the authors can measure the percentage of total re-aiming that is accounted for by explicit adaptation and test how this changes over time. This would answer the question the authors are getting at here.

Figure 4 & 11: Calling subject 4C representative seems problematic. Subjects seems to have a uniform distribution of implicit adaptation between 0 and 20{degree sign}, with two outliers having implicit adaptation near 40{degree sign}. Thus, subject 4C is not necessarily more representative than subject 4B. Perhaps in this and all similar plots, it makes more sense to present the median subject, rather than claiming that anyone is representative?

Line 300: saying that “implicit adaptation was constrained” overstates the case. It was only constrained in some subjects.

Figure 11: some subjects’ implicit adaptation seems appropriate for the mirror reversal; this should be mentioned in the text.

The figure order is switched between the rotation and mirror experiments. This is confusing. To maintain the proper order, the figures for the mirror experiment should be first figure 13, then figure 11, then figure 12, then figure 10.

Typos:

Line 115: made

Line 229: measured

Line 422: underlying

Line 443: admit instead of submit?

Reviewer 2

ADVANCES THE FIELD

The idea of testing for implicit adaptation in the context of a mirror-reversal is interesting. The finding that it is gradually suppressed over 5 days may spark further research. There are also interesting analyses regarding inter-individual differences in implicit adaptation.

STATISTICS

Small sample sizes (n=12) but the authors use caution when interpreting, especially correlations.

COMMENTS

Here the authors report two experiments in which participants (n=12 in each) are exposed to either a 45 deg visuomotor rotation or a mirror-reversal perturbation over 5 consecutive days. They find that for the rotation, implicit adaptation saturates at ∼15 deg and remains stable for each participant across days, but is highly variable across participants. During mirror-reversal implicit adaptation is gradually suppressed over the 5 days.

The work is properly conducted, generally clear and provides novel insight regarding implicit adaptation in the context of visuomotor rotation and mirror-reversal. My main issue is that the work is framed around a debatable working hypothesis that I feel takes away from the novelty of the findings. I have other (minor) comments to ameliorate the paper.

I have a problem with the framing of the paper. It’s unclear why the author assume (or test the hypothesis) that implicit adaptation processes would potentially be making a large contribution to motor skill learning the novo. In my opinion (and to much of the field), there is a pretty clear line between adaptation (a recalibration process of a previously learned movement) and motor skill learning (acquiring an entirely new motor control policy); they are distinct processes that serve different functions (see this recent review for instance: Spampinato and Celnik 2020, Neuroscientist). Even the authors apparently get this (line 47 : “It is difficult to see how asymptotically constrained implicit adaptation could produce long-term motor learning in the absence of explicit re-aiming.”; see also line 426). I do not understand why the authors make a conundrum off of this. Overall this leads the authors to conclude that implicit adaptation cannot account for motor learning de novo, which is quite expected and not very compelling (eg. last sentence of abstract: “implicit adaptation processes, as studied in sensorimotor adaptation paradigms, may only make subtle recalibrations of an existing skill and cannot contribute to motor skill learning de novo” ; or sig statement: “These findings question the utility of implicit adaptation processes to motor skill learning more broadly”). I think this framing reduces the novelty/excitement of the findings. This is perhaps best exemplified on line 511: “we hold that classic implicit adaptation is not the driving force behind motor learning, but that another system is largely responsible.” Sure! Was there actually any current model or theory that entertained this possibility?? If we were to generalize, would the authors also hypothesize that visuomotor adaptation have anything to do with other types of de novo motor skills, like sequence learning? I suggest the authors either bolster their case for why we would expect that implicit adaptation would fully account for de novo motor skill learning (emphasizing more on current literature - and less on 19th century Stratton) or change their premise and focus on the actual results (saturation over 5 days, or decrease in the mirror-reversal). I prefer option 2.

The very large inter-individual differences in implicit adaptation are really intriguing (and almost worrisome for all who rely on this task to learn something robust about the brain!). It would be nice if the authors discussed this more in depth, what this means in terms of the relative interplay between implicit and explicit processes, how it is (un)related to the measured aftereffects, and the broader implications. At current, the discussion is relatively heavy on the explicit process (which was not the main question of the paper) and relatively light on these interesting findings.

I do not understand where the data for each day are in figures 8A and 9A (line 325).

Why isn’t there a figure like figure 3 for the mirror experiment? Also please order the different analyses and figures the same way for both exps. (the aftereffects figure is not at the same place in both).

Minor comments:

Line 35: “dissociated"

Line 115: “made” a reaching movement

Some aspects of the methods are not clear:

Line 118-123: Please present the time allowed for the tapping.

Line 164-170: the target locations are not obvious for the mirror.

The intuition behind the 2 experiments is not obvious by just reading the methods. The first paragraphs of the results for each exp (line 223 and 309) are helpful and could be presented earlier.

## Author Response

1

We would like to thank the reviewers for their thoughtful and detailed comments on the manuscript. We

apologize in advance for some lengthy replies to a few of the comments. The reviewers raised really

interesting questions, which required a great deal of unpacking. We hope that we have clarified a number

of the issues raised in the reviews and have made substantial changes to the manuscript.

Below we address each comment in turn. Reviewer comments are in blue and our response is in black.

Simple changes to the text are italicized and included below. For more extensive changes to the text, we

included page and line numbers that point to the red-lined document.

Synthesis Statement for Author (Required):

SYNTHESIS

The reviewers highlighted the high quality and difficulty of the experiments and recognized the

importance the work in understanding the role of implicit adaptation in motor learning. The reviewers

noted the particular significance of the reduction in implicit learning for the mirror reversal task. Both

reviewers also noted, however, significant issues related to the conclusions that are being drawn from the

results. Both reviewers had major comments about the current exposition, though there is some difference

in perspective about the degree to which adaptation may contribute to de novo motor learning. Both

reviewers were concerned by the large variability across subjects, particularly given the small sample

size, and felt that this variation in behavior was not discussed in sufficient detail. The reviewers provide

detailed suggestions regarding these and other important issues, which you should find useful in revising

your manuscript.

Reviewer 1

ADVANCES THE FIELD

The data collected is difficult to collect because it requires 5 consecutive days of experiments in each

subject. In addition, the experiments seem performed well and data quality seems high.

STATISTICS

A power analysis is required (as detailed in comments to authors) and some error bars are missing in the

plots.

We have included a post-hoc power analysis in the revised manuscript (page 7, lines 115-119) and a

description of our methods is detailed below (on page 5 of this response letter).

SOFTWARE

We downloaded and tried to run Figures_8_9.m but received an error “Unrecognized function or variable

‘plot_format’.”

Apologies for this oversight. It was an in-house function used to format the plots. We have double

checked our code to ensure that there are no more missing contingencies.

COMMENTS

The manuscript explores how practice over several consecutive days impacts implicit adaptation in two

different tasks: a visuomotor rotation task and a mirror task. In the rotation task, implicit adaptation

plateaus after the first day of training and afterwards remains constant. In the mirror task, implicit

adaptation drifts towards 0 throughout the entire practice. The central claim of the paper is that in neither

task does 5 days of training lead to a marked increase in the amount of implicit adaptation. For

visuomotor rotation, the failure of the implicit adaptation to increase is interpreted to mean that implicit

adaptation is limited in its ability to reach full adaptation. In the mirror reversal task, the authors interpret

their result to mean that implicit adaptation has no ability to successfully adapt to mirror reversal. Taken

together, the results lead the authors to question the utility of implicit adaptation for the broader spectrum

of motor skill learning.2

The manuscript targets a key area of exploration in the study of motor control. The authors are correct

that extrapolating findings about implicit adaptation from visuomotor rotation to other motor tasks, such

as the mirror task, can provide valuable insights into the mechanisms of implicit adaptation in general

motor learning. However, we have reservations about the work as it currently stands. These can be

broadly categorized into issues with the experimental design, the statistics, the interpretation of the

existing literature and the interpretation of the results. We detail the major and minor points below.

Major issues

Existing literature:

In discussing the evidence that adaptation only plays a minor role in visuomotor rotations (lines 38-39)

the authors only provide support for a weaker claim: that explicit re-aiming does play a role. Indeed, the

Mazzoni and Krakauer (2006) paper they cite shows implicit adaptation can override explicit strategies.

The authors should also relate to recent evidence showing that long-term retention (in the form of

savings) can actually proceed without formation or recalling of explicit strategies (Yin et al. 2020). We

feel (as discussed below) that the authors need to clarify the point they feel is made by the experiments

and then reassess how their experiments will extend the existing literature. Two additional papers should

also be discussed:

Telgen J Neurosci 2014 (https://www.jneurosci.org/content/34/41/13768)

Yang bioRxiv 2020 (https://www.biorxiv.org/content/10.1101/2020.01.15.906545v1.full)

Based on the reviewer’s comment here and a fresh read of the manuscript, we now see that our narrative

flow (in the introduction) appears to emphasize the existence of explicit re-aiming strategies rather than

discussing how implicit adaptation appears to be limited. We missed an opportunity to discuss several

studies that make the stronger claim that implicit adaptation is limited (note, we don’t think implicit

adaptation plays an insignificant role, rather it can be very useful for small perturbations).

First, when explicit and implicit learning are dissociated using the verbal report procedure, we have

observed that the implicit adaptation asymptotes to a value far less than complete learning and, in fact, the

implicit adaptation function is largely insensitive to rotation magnitude (in this study we tested rotations

of only 15 degrees or larger; Bond and Taylor 2015). Second, when explicit re-aiming strategies are

rendered irrelevant using the visual-error-clamp procedure, a similar pattern emerges: Implicit adaptation

asymptotes too early and appears insensitive to rotation magnitude (tested between 7-95 degrees;

Morehead, Taylor, et al. 2017). We should note that there have been a few modifications to simplicity of

this conclusion, with a few recent reports showing that implicit adaptation is proportional for rotations

under approximately 8 degrees and there may be an effect of reward (Kim, Morehead et al., 2018; Kim et

al., 2019; Leow et al., 2018; Hutter and Taylor 2018). Finally, a recent study by Avraham, Morehead, et

al., 2021 suggests that the contribution of implicit adaptation appears to even decrease with extended

training, similar to the result we observed here.

We have now expanded our discussion of these studies in the introduction (page 3, lines 44-52) to provide

more support for the notion that implicit adaptation may be limited:

"In parallel to the recognition that strategies play an important role in adaptation tasks, we see rapidly

emerging evidence that the implicit process is highly stereotyped regardless of the particular task

demands. The implicit process asymptotes to a value far less than complete learning and is largely

insensitive to perturbation magnitude (Bond and Taylor, 2015; Morehead et al., 2017). Morehead and

colleagues (2017), revealed a stereotyped implicit learning curve when the incentive to strategize was

completely removed. Surprisingly, asymptotic implicit learning remained insufficient to account for the

perturbation after many hours of practice. (Morehead and Smith, 2017, Morehead et al., 2017; Kim et al.,3

2018). Finally, recent evident suggests that the contribution of the implicit process decreases with

extended training (Avraham, et al., 2020).”

The reviewer also noted a few studies that they would like us to address. We feel that the title of Mazzoni

and Krakauer (2006) can be a bit misleading. In that study, while the explicit re-aiming strategy is

initially effective, implicit adaptation gradually begins to accumulate causing performance to deteriorate.

Despite their title, it doesn’t necessarily mean that implicit adaptation completely overrides the strategy,

but instead is simply added on top of the aiming strategy. We’ve run an extended version of that study (4

times as many trials) and found that as implicit adaptation causes more and more deterioration in

performance, participants will begin to explicitly re-aim their movements to offset implicit adaptation

(Taylor and Ivry 2011).

The savings-in-relearning study by Yin and colleagues (2020) is difficult to interpret as savings in the relearning block could be the result of an explicit strategy. Yes, they used conditions to prevent or

discourage strategy use in the first rotation block, using either an error clamp or gradual perturbations, but

the washout and re-learning blocks involved abrupt rotations. Thus, the participants could have formed a

strategy in the re-learning block or even had meta-learning/structural learning of a strategy during the

washout (Bond and Taylor 2017). Or, specific to the error clamp condition, the participants could have

observed the pattern of errors during the clamp block and used that information to quickly form a strategy

in the re-learning block (McGregor & Gribble 2015, 2017, Wanda, Li, Thoroughman 2013). Furthermore,

specific to the gradual condition, gradual conditions do not always imply that participants didn’t use a

strategy. We have observed explicit re-aiming in the later stages of a gradual condition, presumably

because the rate of gradual increase was too high for implicit adaptation to keep up or implicit reached

asymptote (Butcher, Ivry et al., 2018). We should also note that Morehead et al (2015) and Haith et al

(2015) did not observe what would be considered implicit savings, and Avraham et al., 2021 suggests that

implicit is actually reduced in the re-learning block, and even performed a post-hoc analysis on the

dataset from Yin and Wei 2020 and found that those data show attenuation of implicit adaptation too. We

should note that these findings have been challenged by Albert et al., 2021, so clearly more work is

required to flesh out all of these possibilities.

Finally, the study by Yang et al. 2020 may suggest that under a more de novo learning situation - one

that requires continuous feedback control, the feedback controller may become tuned to a mirror reversal

(revealed by their control systems approach). While feedback control was examined in Telgen et al.

2014, Kasuga et al. 2015, Grisenko and Kalaska 2009, and Hadjiosif et al., 2021, these tasks were center out reaching tasks and may have emphasized feedforward over feedback control, and may not necessarily

be a de novo learning situation per se. We now discuss these studies in detail in our discussion.

We have made substantial changes to the introduction, pages 2-6, and discussion, pages 29-35, to address

these issues (since the changes are substantial, we did not include the text in this response letter).

Experimental design:

The authors set out their question in the introduction: can implicit adaptation lead to long term motor

learning in the absence of explicit re-aiming. The experimental design does not seem to answer this

question. There is simply no condition with an absence of explicit re-aiming. When this question has been

asked (see Yin 2020 referenced above), it has been answered in the affirmative. Again, the authors should

clarify which question their data addresses and make a strong link between the question and the

experimental design.4

Apologies for the lack of clarity on our part, our goal was not to determine if implicit adaptation would

lead to long-term learning in the absence of explicit re-aiming. Instead, we wanted to know if implicit

adaptation would begin to replace explicit re-aiming.

We have included the sentence (page 4, lines 68-70): “If the implicit process, as measured in this task,

will eventually contribute substantially to overall compensation, we expect to see a steady increase in the

proportion of learning accounted for by the implicit process over time.

Indeed, the experimental design seems to encourage explicit re-aiming. For example, it has been shown

previously that reporting can affect different aspects of adaptation: it promotes full asymptote (see

preprint by Langsdorf et al. 2020), it has been associated with more explicit re-aiming and less implicit

adaptation (see Leow et al. 2018 and preprint by Maresch et al. 2020). Moreover, large rotations (Bond et

al. 2015) that are introduced abruptly (Werner et al. 2015) also cause more explicit re-aiming. This

suggests that possibility that the authors consider collecting data in conditions which are known to

contribute to stronger implicit learning. At the least, this should be discussed as a limitation of this study.

We agree with the reviewer that our experiment design encourages participants to explicitly re-aim - we

require them to report their aim on each trial. When participants are instructed to indicate their aim on

each trial (regardless of the reporting method: verbal reports, adjusting a report using a keyboard, mouse,

touch screen, etc.) the rate of performance improvements seems to be faster compared to groups that are

not required to report (more standard rotation tasks). However, when the aftereffects of report groups are

compared to non-reporting groups, there is very little difference (Taylor et al 2014; Bond and Taylor

2017). Interestingly, Maresch and Donchin (2020) found similar sized aftereffects across their groups,

which were also far less than the size of the rotation (∼35 degrees on average for a 60-degree rotation),

although they found more radical differences between groups when assaying explicit/implicit during

training. To our knowledge, any study that instructed participants to stop re-aiming during washout has

not revealed aftereffects that have fully compensated for the rotation. The visual error-clamp procedure

reinforces these results, as re-aiming is likely not at play and yet implicit adaptation does not fully

compensate for the rotation (Morehead et al., 2017; Kim et al., 2019).

Again, however, our goal was to determine if implicit adaptation would eventually counteract a

reasonably large rotation - one that typically causes participants to explicitly re-aim - given sufficient

training. It should be noted that we don’t know how to define or operationalize “more” or “less” explicit

re-aiming. Yes, participant’s explicit re-aiming angle can be larger or smaller but that doesn’t necessarily

mean that it is more or less explicit. A recent paper has shown that two different explicit re-aiming

strategies for the same sized rotation, one likely relied on mental rotation of a planned movement vector

while the other reflected retrieval of a cached motor plan (McDougle and Taylor 2019). The former

requires longer preparation time to implement, but here too it is difficult to say if this is more or less

explicit. Going forward, we will need figure out how to operationalize these terms as more or less explicit

knowledge, deliberation time, etc.

Along similar lines, the authors argue (line 44) that because implicit adaptation is only partial, it cannot

lead to long-term motor learning. This seems like a strange claim. Can it be justified? The claim that the

authors can make on the basis of this design is that not all explicit learning eventually becomes implicit.

This seems like a straw man argument. Even if it is taken seriously, it may be that more than 5 days are

necessary to make some tasks fully implicit. The Stratton (1896) and Kohler (1941) studies which led the

authors to choose 5 days actually used five full days of continuous exposure. This is much more exposure

than used in the current study. We imagine that extending the experiments to more than 5 days or trying

to have subjects practice for entire days will not be practical, but this limitation does need to be discussed.5

We entirely agree with the reviewer’s assessment here. It may very well be the case that much more

exposure is required to develop a fully implicit mapping. While one hour a day for five days may not be

sufficient to have an implicit mapping that fully accounts for the rotation, we were expecting to see a

significant positive slope of implicit adaptation across the days. That is, we expected to see implicit

adaptation gradually rise while explicit re-aiming gradually decrease. On average, we don’t see an

increase and even a slight decrease in implicit with more exposure, which has recently been reported by

Avraham and colleagues (2021). So, yes, full implicit adaptation may be a straw man, but a gradual

increase in implicit adaptation was what we were expecting at a minimum.

We have modified the text to reflect this more modest prediction on page 4, lines 67-72:

"In Experiment One, we investigate adaptative learning to a visual perturbation over five days of

training. If the implicit process, as measured in this task, will eventually contribute substantially to

overall compensation, we expect to see a steady increase in the proportion of learning accounted for by

the implicit process over time. We find that the implicit process, on average, does not change over the

training period. However, we also find substantial between-subject differences that raise questions about

the validity of the measure.”

Statistics:

It would be helpful to present a power analysis that would show that this sample size is large enough to

show an effect of at least 2 - 3 degrees. If not, the authors should consider collecting additional data to

achieve 90% power for an effect size of 2-3 degrees. In this case, many would argue that the additional

subjects should be analyzed separately in order to preserve the purity of the statistical approach. The

power of the original study for a small effect size should be reported.

We are unsure for what data the reviewer would like to see a power analysis for 2-3 degrees of change.

We have taken the comment to refer to the t-tests applied to the difference between the first and last days

of implicit adaptation (please correct us here if we misinterpreted the comment). But assuming this was

the case, we were hesitant to run a power analysis, in part, because of the straw man comment above. If

we were expecting adaptation to be full (∼45 degrees) after five days and we knew that implicit

adaptation asymptotes ∼20-25 degrees from previous work, then our power would be unreasonably high

(∼2 subjects). If we took the more modest prediction, that implicit would simply increase between days,

then we don’t know what effect size to estimate as no study has examined implicit adaptation across days

(using methods that attempt to dissociate or isolate implicit adaptation). 2-3 degrees, as the reviewer

suggests, sounds reasonable but is ultimately arbitrary. As such, we opted to recruit a sample size that is

in the range of many studies of visuomotor rotations (10-15 subjects).

However, assuming a 3-degree change in mean and a SD of 3-degrees results in a suggested sample of 10

participants to achieve 90% power. A post-hoc power analysis results in a power level of just over 85% to

observe the reduction of implicit adaptation found in Experiment 2.

We have now added this to the methods (page 7, lines 115-119) with the caveat is that we didn’t know a

priori what effect size to estimate.

"We did not have an a priori effect size estimate with which to calculate a power analysis. However,

assuming a 3-degree change in mean and a SD of 3-degrees results in a suggested sample of 10

participants to achieve 90% power. A post-hoc power analysis conducted on the reduction of implicit

adaptation found in Experiment 2 results in a power level of just over 85% to observe the reported

effect.”6

Figures 6 & 10: Error bars on the bar for the mean and individual subject error bars would be helpful.

Also, a scatterplot of implicit adaptation vs aftereffect with error bars in both directions would give a

better indication of how these measures relate to each other.

Figures 6 and 10 (now 6 and 12) were changed as suggested. Similar to Maresch and Donchin, we too

observe a disconnect between implicit adaptation measured via subtraction during training and with

aftereffects. However, we should note that neither measure shows full implicit adaptation (∼45 degrees)

after 5 days of training.

Figure 6. (A) Average aftereffect for each subject averaged across the 32 trials of no-feedback washout

(with subjects instructed to aim and move directly to the target). Error bars represent one standard error

of the mean. (B) The time course of washout for the average across subjects, and individual trial values

for the three example subjects defined in Figure 3. Shaded area represents between-subjects standard

error. (C) Correlation between each individuals’ adaptation as measured via subtraction (hand angle

minus explicit re-aiming over the last 32 trials of training) vs washout (average hand angle in 32 washout

trials). Error bars represent one standard error of the mean along each dimension. The marker color and

shape represent an individual subject across all figures in this manuscript.7

Figure 12. (A) Average corrected aftereffects for each subject across the 32 trials of no-feedback,

washout (with subjects instructed to aim and move directly to the target). Each subject is jittered left to

right in order of least to most explicit re-aiming at the end of Day 5. (B) The time course of washout for

the average across subjects and individual trial values for three representative subjects [#5 (Lowest), #8

(Median), #12 (Highest), re-aiming on last day). The shaded area represents the standard error of the

mean for all subjects.

Lines 328 - 329: if arguing for similarity, an equivalence test should be conducted (Lakens Advances in

Methods and Practices in Psychological Science 2018 has a nice tutorial).

The reviewer asks us to determine the threshold for an effect that is “trivially small”. The equivalence test

requires positive and negative bounds against which we could test the result but given the exploratory

nature of this data and analysis there is no particular reason to pick {plus minus}1{degree sign} over {plus minus}5{degree sign} or {plus minus}10{degree sign}. Although we do

not believe this arbitrary determination will provide additional information beyond the simple t-test, we

conducted the suggested equivalence test against a bound of {plus minus}6{degree sign} (conservatively proposing that anything

more than a 1/4 reduction of implicit recalibration is nontrivial). The result was consistent with our

previous suggestion that implicit recalibration was suppressed in the mirror-reversal task (t=3.2, p<.01).

Figure 10: this figure is meant to show that aftereffects are in the direction opposite to the appropriate

direction, suggesting an inability of subjects to change the way their implicit system corrects. This

argument would be made more powerful if the results were broken down by quadrants, to show that for

each the corrections were in the “standard” and not the “reversed” direction.8

We have added additional panels to figure 10 (now 12) to show the difference in aftereffects for different

quadrants in the mirror task. See above.

Discussion:

The discussion, and the paper generally, feels to us that it glosses over the inter-subject differences.

Individual subject variability was quite pronounced: some subjects show almost 45{degree sign} of

implicit adaptation and others close to 0{degree sign} in the rotation task. The sample size is very small

and the data is very noisy (as shown by the large between-subject variance). In line 233, the SEM of reaiming and implicit adaptation is around 16{degree sign}, meaning implicit adaptation could be as big as

explicit re-aiming in some cases. In the mirror task, some subjects show implicit adaptation appropriate

for the mirror task and others do not. This is especially important since task details could affect the

proportions of subjects showing more implicit adaptation in the two tasks. The discussion should fully

address both intersubject variability and the possibility that subtle details of task design may be

influencing the results.

We agree that the sample size is quite small and that individuals show substantial differences in the

relative contribution of implicit and explicit re-aiming. This is why we carefully included many examples

of different subjects in figures, so that the reader could view the full gambit of learning styles. However,

we do not think that additional subjects would significantly alter the results - as you would simply see

more examples of the various ‘types’ of subjects. Additional subjects would not change the fact that there

is great variability between subjects.

We conducted a meta-analysis across 5 studies, with over 50 subjects, using the aim-report procedure

(identical to Taylor, Krakauer, and Ivry 2014). It shows the same variance as seen in these experiments.

While there are a few subjects that appear to show full implicit learning, the vast majority appear similar

or less to the mean observed in the present study. We opted not to include this meta-analysis in the paper

given the differences in study design, such as number of trials and verbal reports, as well as difficult in

visualizing learning time courses for all 50 subjects. Nonetheless, we thought the reviewer may find the

data interesting.

Figure: A) Final hand angle (purple), verbally reported aiming angle (explicit, blue) and subtraction

(implicit, red). B) The time series was fit with a two-state model fitting, showing the variety of explicit

and implicit learning time courses. Rotation was present between trials 57-376 (vertical dashed lines). In

washout, participants were instructed to stop aiming and cursor feedback was removed.9

Results (page 23, lines 371-376):

"Analysis of within- and between-subject variance: As with the visuomotor rotation, there was significant

variance in how subjects responded to the visuomotor mirror reversal (approximately -15{degree sign} to 15{degree sign}, Figure

11). Subjects were successful through a combination of implicit adaptation and explicit re-aiming, lost all

implicit adaptation by the end of training, or reversed the direction of implicit adaptation. Reversed,

visualized as negative, implicit adaptation is appropriate for the mirror reversal task.”

Discussion (page 30, lines 468-476):

The implicit process in the mirror reversal task initially was counterproductive to performance before

becoming suppressed with increased training. These findings may have been expected given the recent

evidence that the implicit process falls well short of full learning in visuomotor adaptation tasks

(Morehead and Smith, 2017; Morehead, Taylor, Parvin, and Ivry, 2017; Kim et al., 2018; Kim, Parvin,

and Ivry, 2019) and in the wrong direction in the mirror reversal task (Gritsenko and Kalaska 2009;

Lilicrap et al 2013; Telgen 2014; Kasuga 2015; Hadjiosif et al., 2021). Nonetheless, we thought that this

limitation of implicit process may be overcome with more training, an idea that failed to be supported by

our five-day experiments.

The discussion should address the authors expectation (lines 331-334) that implicit adaptation should

become more appropriate for the mirror reversal task after more days of training. What are the two

alternative theories of how implicit adaptation might work with regards to the mirror task and how do

they lead to different expected results. Once this is discussed, the actual results can be understood in the

correct context.

We now discuss on pages 32-33 (lines, 516-550) how implicit adaptation may become more appropriate

either by changing the error derivative or reparameterization of the transformation matrix:

"Implicit recalibration in the mirror reversal task

The mirror reversal results present an exciting future opportunity to determine the error signal

that initiates suppression of the implicit process in the mirror reversal task. Large variability in

individual behavior provides a foothold to understanding suppression of implicit processes. Here we

consider a few possible explanations that could be a target for future research.

The mirror reversal, unlike a rotation task, has an error-signal with a constantly switching sign.

Previous work has shown that inconsistency in error signals reduces overall learning (Castro et al.,

2014) and the implicit process in particular (Hutter and Taylor, 2018), which is consistent with the motor

system storing a history of errors (Herzfeld et al., 2014). Here, we would expect participants that

experienced more error switching, through their unique history of missing the target, to suppress implicit

adaptation more quickly and fully (Albert et al., 2020).

Second, the difference between rotations and mirror-reversals could be thought of as a

reparameterization, as previously considered in rotations, compared with learning a completely new

structure, suggested for the mirror reversal (Braun, Aertsen, and Wolpert, 2009). The reparameterization

of the transformation matrix, as seen in adaptation to a rotation, might be the fundamental function of the

implicit process. Indeed, the implicit process may be exceptional at matrix reparameterization. In

contrast, learning a mirror reversal requires learning the full structure of the task, including the

relationship between parameters in the transformation matrix. It appears that the implicit process tries to

reparametrize the transformation matrix as if it were a rotation even if doing so is inappropriate for the

task. Only after days of training does the implicit process appear to stop reparametrizing inappropriately.

It could be that once a new structure is specified, the structure of the mirror reversal, the implicit process

becomes appropriate for this new structure. The speed of learning this structure would dictate how

quickly implicit processes began reparametrizing appropriately.10

A slightly lower-level of description of this problem, one rooted in control engineering, was put

forth in a recent study by Hadjiosif and colleagues (2021), which compared adaptation under a

visuomotor rotation and a mirror reversal as a way to distinguish between updating forward model

updating and updating a control policy. They too find that the implicit process in a mirror reversal was

directed in the wrong direction to counter a mirror reversal, which is inconsistent with the updating a

forward model. Instead, implicit learning of a mirror reversal may require direct updating of a control

policy. Learning a new control policy would again have the outcome of changing the structure of the

transformation matrix.

These explanations are highly speculative, and additional research is necessary to pin down the

reason for implicit processes being counterproductive in a mirror reversal task, and the subsequent

suppression of implicit recalibration.”

Much of the discussion (lines 443-512) is devoted to the topic of proceduralization and theories of motor

learning. This seems off topic both in terms of the central question in the manuscript and the design of the

experiments. It also seems largely speculative. We recommend reducing this part of the discussion to no

more than 20 lines (slightly less than a page).

We have significantly reduced the proportion of the discussion that is dedicated to the topic of

proceduralization. We do believe it is relevant and should be discussed in light of our results, especially

given recent resurging interest in this topic. It has now been reduced to ∼35 lines of text.

There is no clearly set out discussion of the studies limitations. This should be added. It should discuss, at

the least, the intersubject variability, the relatively small number of subjects, the relatively limited amount

of training in the tasks, and the interpretational difficulties associated with measures of explicit and

implicit adaptation.

We have addressed this concern in various places throughout the manuscript, particularly in the

discussion on pages 29-35. We made sure to highlight potential reasons for the high degree of between subject variability we observed and differences in reporting measure versus aftereffects.

Minor comments

Line 44: asymptotic adaptation remained partial instead of insufficient?

The authors’ continuous implicit measure is solely derived from explicit reports. They also use aftereffects

as a measure of the final implicit adaptation. This can be problematic since these measures have been

found to differ profoundly in many situations (Taylor et al. 2014, Bond et al. 2015, Leow et al. 2015,

Bromberg et al. 2019). The authors themselves find a mismatch between these two measures. It has been

suggested that implicit adaptation as derived from reporting may not measure the same as the aftereffect

(see preprint by Maresch et al. 2020). This mismatch between measures should be addressed in the

discussion of possible limitations of the study.

Following up on our response above, we agree that it would be good to discuss this issue in the

manuscript. An entire section entitled “Limitation of the Implicit Process and Measurement Reliability"

has been added to the discussion, in particular (page 31, lines 493-509):

"An additional possibility for the limited degree of implicit recalibration observed here, as well as the

variability between participants, may be the result of explicit strategy use. While several studies

suggested a considerable degree of independence between implicit and explicit processes (Mazzoni and

Krakauer 2006; Taylor and Ivry 2011; Taylor et al., 2014), this has recently been challenged.

Participants who reported their explicit aiming direction throughout training, showed less implicit11

recalibration than participants who were only probed at the end of training (Maresch and Donchin

2020). By asking participants to report their aiming location on each trial, learning may be shifted more

toward explicit strategies. It should be noted that in the original study of the aim report method, there

was very little difference in the size of aftereffects between groups that reported on each trial versus never

reported (Taylor et al., 2014). This was, in part, our reasoning for not including a no-report control

group in conjunction with the difficulty of recruiting participants for a five-day study where the primary

dependent measure (i.e., aftereffects) occurs only once at the end of the study. What’s more, like Maresch

and Donchin (2020), we too find no correlation between the degree of implicit adaptation measured via a

subtraction measure during training and the size of the aftereffect. Nonetheless, implicit adaptation

appeared limited in both measures. Clearly, more work is needed to flesh out this psychological

equivalent of the measurement problem.”

Many analyses are not described in the methods (individual subject differences, regression comparing

adaptation at the end of each day to beginning of next day, comparison between implicit and aftereffect

on day 5). These should be included in the methods section. Was online or endpoint feedback used during

rotation trials? The description is somewhat confusing: line 125: a small circular cursor (0.15 cm 125

radius) provided online feedback and then line 136: endpoint feedback was displayed for 500 ms.

Apologies for the lack of clarity here. There was both online and endpoint feedback provided as follows:

Online feedback was provided throughout the reach, but once the hand past 7 cm (the target distance), the

cursor was frozen at the position for 500 ms (endpoint).

Page 9, lines 159-164:

"During each trial, once the right hand moved out past 7 cm, endpoint feedback was displayed for 500

ms in a position contiguous with the last point of on-line feedback. If the position of the cursor completely

overlapped with the target (< 1 deg. of angular deviation), the subject heard a pleasant “ding”.

Otherwise, an unpleasant “buzz” sounded. The feedback “Too Slow” was given if the reach time from

start to 7 cm exceeded 600 ms (<1% of trials, these trials were excluded from further analysis).”

Is there a reason that 12 targets were used in Experiment 1? How many targets in Experiment 2 (I think 4,

but it’s not explicitly stated)?

We used 16 targets in experiment one to cover the full learning space. Since experiment 1 used a 45

degree rotation, we wanted to replicate the same size perturbation in the mirror reversal task of

experiment 2. Target location in the mirror task defines the size of the perturbation. For example, a target

at 0 degrees (in a standard Cartesian) results in a 180 degree perturbation in the mirror task. Likewise, a

target at 67.5 degrees results in a 45 degree perturbation (hand goes to 67.5 and cursor is reflected across

the y-axis and goes toward 112.5 degrees). Given this constraint, we could only have 4 targets to provide

a consistent 45 degree perturbation.

Page 10-11, lines 193-197:

"In order to allow comparison between the two experiments, the four targets in Experiment Two (Mirror Task) were located 22.5{degree sign} from the midline so that the solution to the perturbed reach was a 45{degree sign} angle.

The 12 targets in Experiment One were evenly spaced 22.5{degree sign} apart on the aiming ring (Figure 2). Both

experiments were programmed such that each of the targets appeared before any was again repeated.”

Reaction time calculation line 186-187: Reaction times were calculated as the interval from target

appearance to aim-report, except where noted. The authors call the “re-aiming reaction time”. While this

seems appropriate, I’m still wondering in how far this really reflects an actual reaction time. A short12

discussion about what this type of reaction time means and how it differs with other reaction time

calculations would be desirable.

In full disclosure, we didn’t design the study with reaction times in mind and participants were not under

any time pressure, so our reaction time measures should be viewed with caution. We don’t feel

comfortable speculating as to the number of processes that could be subsumed under this measurement

(fixating the target, selecting an aim location, tapping the touchscreen, planning the movement with the

right hand, etc.).

Page 8, lines146-148:

"Subjects were instructed to proceed through the experiment as quickly as possible, however, there was

no time limit for tapping the aim location or beginning the reaching movement.”

Line 233: How is the SEM calculated? Did the authors account for within subject variability?

Our SEM calculation is only between subject variability. We found the square of the difference between

each data point (subject) and the sample mean, finding the sum of those values. Then, that sum was

divided by the sample size minus one (variance). Taking the square root of this variance results in the SD.

Finally, we divide the SD by the square root of the sample size (12) to give us SEM.

Line 235-237: the authors are correct that a direct comparison of explicit and implicit is inappropriate.

However, the authors can measure the percentage of total re-aiming that is accounted for by explicit

adaptation and test how this changes over time. This would answer the question the authors are getting at

here.

Our intent here was simply to report the explicit and implicit values, but we didn’t want a reader to think

that we should do any comparisons between explicit and implicit since implicit is defined as hand angle

minus implicit.

Figure 4 & 11: Calling subject 4C representative seems problematic. Subjects seems to have a uniform

distribution of implicit adaptation between 0 and 20{degree sign}, with two outliers having implicit

adaptation near 40{degree sign}. Thus, subject 4C is not necessarily more representative than subject 4B.

Perhaps in this and all similar plots, it makes more sense to present the median subject, rather than

claiming that anyone is representative?

Apologies for the confusion here. Our use of the word “representative” in the figure caption “A subject

(#6) representative of the average taken from the middle of the distribution,” was a poor choice in

wording. We were purposely avoiding the “representative” subject. Instead, we were trying to illustrate

the diversity in adaptation curves across the group. We rank ordered the subjects in terms of implicit

adaptation (Fig. 4A), then showed the subject with the least (Fig. 4B), median (Fig. 4C), and most

implicit adaptation (Fig 4D). Our use of representative here was meant to simply mean that the subject in

the middle is the median subject and represents the implicit value observed in the average. We have

removed the word representative entirely.

Line 300: saying that “implicit adaptation was constrained” overstates the case. It was only constrained in

some subjects.

The wording has been adjusted as follows on page 19, lines 331-334:

"On average, we found the implicit process to be constrained, which is consistent with the findings from

studies examining the capacity of single-session implicit learning. However, we saw great variability13

between subjects, including evidence from two subjects that implicit recalibration could eventually

replace explicit re-aiming.”

Figure 11: some subjects’ implicit adaptation seems appropriate for the mirror reversal; this should be

mentioned in the text.

The following is included in the text on page 23, lines 371-376:

"As with the visuomotor rotation, there was significant variance in how subjects responded to the

visuomotor mirror reversal (approximately -15{degree sign} to 15{degree sign}, Figure 11). Subjects were successful through a

combination of implicit adaptation and explicit re-aiming, lost all implicit adaptation by the end of

training, or reversed the direction of implicit adaptation. Reversed, visualized as negative, implicit

adaptation is appropriate for the mirror reversal task.”

The figure order is switched between the rotation and mirror experiments. This is confusing. To maintain

the proper order, the figures for the mirror experiment should be first figure 13, then figure 11, then figure

12, then figure 10.

Thank you for pointing this out. We have rearranged the figures as suggested.

Typos:

Line 115: made

Line 229: measured

Line 422: underlying

Line 443: admit instead of submit?

The typos have been fixed.

Reviewer 2

ADVANCES THE FIELD

The idea of testing for implicit adaptation in the context of a mirror-reversal is interesting. The finding

that it is gradually suppressed over 5 days may spark further research. There are also interesting analyses

regarding inter-individual differences in implicit adaptation.

STATISTICS

Small sample sizes (n=12) but the authors use caution when interpreting, especially correlations.

We agree that a sample size of 12 subjects is on the smaller side. As discussed above (on page 5 of this

response letter), an a priori power analysis was too optimistic (∼2 subjects). As such, we opted to recruit a

sample size that is typical of visuomotor adaptation tasks (10-15 subjects). While we observed quite a

range of variation between subjects, an individual’s behavior was much more consistent across days, and

we tried to be very transparent about the diversity of behaviors between subjects. In the end, we don’t

think that recruiting more participants would radically change the results. As the reviewer noted, we made

sure to be extra cautious in interpreting the results.

COMMENTS

Here the authors report two experiments in which participants (n=12 in each) are exposed to either a 45

deg visuomotor rotation or a mirror-reversal perturbation over 5 consecutive days. They find that for the14

rotation, implicit adaptation saturates at ∼15 deg and remains stable for each participant across days, but

is highly variable across participants. During mirror-reversal implicit adaptation is gradually suppressed

over the 5 days.

The work is properly conducted, generally clear and provides novel insight regarding implicit adaptation

in the context of visuomotor rotation and mirror-reversal. My main issue is that the work is framed around

a debatable working hypothesis that I feel takes away from the novelty of the findings. I have other

(minor) comments to ameliorate the paper.

I have a problem with the framing of the paper. It’s unclear why the author assume (or test the hypothesis)

that implicit adaptation processes would potentially be making a large contribution to motor skill learning

the novo. In my opinion (and to much of the field), there is a pretty clear line between adaptation (a

recalibration process of a previously learned movement) and motor skill learning (acquiring an entirely

new motor control policy); they are distinct processes that serve different functions (see this recent review

for instance: Spampinato and Celnik 2020, Neuroscientist). Even the authors apparently get this (line 47 :

"It is difficult to see how asymptotically constrained implicit adaptation could produce long-term motor

learning in the absence of explicit re-aiming.”; see also line 426). I do not understand why the authors

make a conundrum off of this. Overall this leads the authors to conclude that implicit adaptation cannot

account for motor learning de novo, which is quite expected and not very compelling (eg. last sentence of

abstract: “implicit adaptation processes, as studied in sensorimotor adaptation paradigms, may only make

subtle recalibrations of an existing skill and cannot contribute to motor skill learning de novo” ; or sig

statement: “These findings question the utility of implicit adaptation processes to motor skill learning

more broadly”). I think this framing reduces the novelty/excitement of the findings. This is perhaps best

exemplified on line 511: “we hold that classic implicit adaptation is not the driving force behind motor

learning, but that another system is largely responsible.” Sure! Was there actually any current model or

theory that entertained this possibility?? If we were to generalize, would the authors also hypothesize that

visuomotor adaptation have anything to do with other types of de novo motor skills, like sequence

learning? I suggest the authors either bolster their case for why we would expect that implicit adaptation

would fully account for de novo motor skill learning (emphasizing more on current literature - and less on

19th century Stratton) or change their premise and focus on the actual results (saturation over 5 days, or

decrease in the mirror-reversal). I prefer option 2.

We feel that this is a very tricky issue to address. We (obviously) agree with the reviewer that implicit

adaptation appears to be very different from de novo motor skill learning and this view has gained

attraction in recent years (as pointed out in reviews by Spampinato and Celnik 2020 and Krakauer et al.,

2019). However, in many of the foundational papers that motivated the internal model adaptation

framework, it has been suggested that adaptation of an internal model formed a critical aspect of motor

skill learning. And sensorimotor adaptation tasks have been viewed as studying internal model adaptation

(what we believe would be viewed as implicit adaptation). Furthermore, it is very often the case that a

study is motivated by describing skill learning and then there’s a switch to studying a sensorimotor

adaptation task (the senior author here is likely guilty of this very “bait-and-switch”).

Just a quick look through some “foundational” papers for the internal model framework:

Jordan and Rumelhart (1992) setup their paper saying “Consider a skill-learning task such as that faced by

a basketball player learning to shoot a basket” and then proceed to build-up the internal model

framework. Cognitive Science.

Mussa-Ivaldi (1999): “The acquisition of complex skills is facilitated by internal models of limb

dynamics”. Current Opinion Neurobiology.15

Wolpert, Ghahramni, and Flanagan (2001): “Skilled motor behaviour requires both inverse and forward

internal models. Motor learning can be viewed as the acquisition of forward and inverse internal models

appropriate for different tasks and environments.” Trends in Cognitive Science.

Shmuelof et al., (2011) takes a more nuanced view, but includes adaptation as a mechanism of skill

learning: “We propose that motor skills are acquired through the combination of fast adaptive processes

and slower reinforcement processes.” Neuron. We note that this group has carved off adaptation as a

process unrelated to skill learning now and, in fact, recently published in The Journal of Neuroscience

entitled “Did we get sensorimotor adaptation wrong? Implicit adaptation as direct policy updating rather

than forward-model-based learning” clearly challenging aspects of the internal model framework.

However, we cannot be absolutely sure if the authors held the view that studying adaptation tasks was a

model for motor skill learning at the time of writing these papers. Furthermore, given the conviction of

the reviewer that no one really held this view and our agreement that implicit adaptation cannot form the

basis of skills, we have reframed the paper to take a view that is at least consistent with adaptation

forming part of a motor skill execution - this may be a more reasonable, consensus view. Nevertheless,

our findings are still problematic for this view, as implicit adaptation can work in opposition to task

performance and appears to be limited on average.

We have reframed the manuscript with this view throughout.

Abstract

These findings are consistent with a handful of recent studies suggesting that implicit adaptation

processes, as studied in sensorimotor adaptation paradigms, only make subtle recalibrations of an existing

skill and cannot contribute to motor skill learning de novo. However, individuals solve the task in a

highly heterogeneous fashion, with some compensating using implicit adaptation and others relying on

explicit re-aiming. This heterogeneity calls into question the usefulness of using group-level data when

analyzing explicit re-aiming and implicit adaptation in visuomotor adaptation tasks.

We agree with the reviewer, looking at only the averages can be highly misleading and doesn’t reflect the

diversity of the study population. This is why we presented data from individual subjects that spanned the

"distribution” in addition to the mean. We have now included a meta-analysis from a number of data sets

from our lab examining the dissociation between explicit and implicit learning in visuomotor rotation task

(page 8, above). Again, we observe a similar level of heterogeneity at the individual level.

We should note that we don’t think that individuals are volitionally choosing to solve the task differently

but given their own personal limitations in implicit adaptation may need to have a larger proportion of the

rotation compensated for through an explicit re-aiming strategy.

We have underscored this problem of averaging in our discussion (page 35, lines 593-594): “This

variation questions the validity of averaging over subjects to make claims about implicit processes"

Significance Statement

In this set of studies, we find that implicit adaptation cannot fully account for learning in adaptation tasks,

such as the visuomotor rotation and mirror reversal tasks, even following several days of training. In fact,

implicit adaptation can be counterproductive to learning. These findings question the utility of implicit

adaptation processes to motor skill learning more broadly. However, we find significant between subject

differences that call into question traditional interpretation of these group-level results.

The very large inter-individual differences in implicit adaptation are really intriguing (and almost

worrisome for all who rely on this task to learn something robust about the brain!). It would be nice if the16

authors discussed this more in depth, what this means in terms of the relative interplay between implicit

and explicit processes, how it is (un)related to the measured aftereffects, and the broader implications. At

current, the discussion is relatively heavy on the explicit process (which was not the main question of the

paper) and relatively light on these interesting findings.

We hope that our inclusion of the meta-analysis (page 8, above) and resulting discussion of the individual

differences has helped bring attention to this issue.

The following is included in the discussion on page 30, lines 479-486:

"Enormous variability, spanning the entire range from zero to full adaptation through implicit processes,

was seen across individuals. This suggests that the traditional method of averaging over participants is

simplifying, and perhaps obscuring, experimental results (Gallistel 2004). It remains an open question as

to why there is such a degree of inter-subject variability; however, this degree of variability has been

observed in several studies where for even small rotations, on the order of 3-10{degree sign}, they found implicit

recalibration ranging from nearly zero to almost 50{degree sign} (Stark-Inbar et al., 2017; Morehead et al 2107; Kim

et al., 2018; Kim et al., 2019).”

I do not understand where the data for each day are in figures 8A and 9A (line 325).

The x-axis was confusingly labeled by bin number. It has been adjusted to indicate days.

Why isn’t there a figure like figure 3 for the mirror experiment? Also please order the different analyses

and figures the same way for both exps. (the aftereffects figure is not at the same place in both).

Rotating all the targets to a common axis in the mirror reversal task isn’t as straightforward as it is in a

visuomotor rotation task. The mirror reversal task is effectively like a dual adaptation task where different

targets have different rotation directions applied (CCW in quadrant 1, CW in quadrant 2, CCW in

quadrant 3, and CW in quadrant 4). This is why we opted to both include the state-space model

predictions and keep the data separated for the quadrants with common directions as if it were a rotation

task. Of course, we can do extra folds of the quadrants and flipping signs to get onto a common axis, but

we felt that this becomes much less transparent. Below is an example of the data all rotated and flipped to

a common axis, but we would prefer not to include this in the manuscript unless the reviewer has a strong

preference for it.17

The ordering of figures has been changed to be more symmetrical with experiment 1.

Minor comments:

Line 35: “dissociated"

Line 115: “made” a reaching movement

Typos fixed.

Some aspects of the methods are not clear:

Line 118-123: Please present the time allowed for the tapping.

In full disclosure, we didn’t design the study with reaction times in mind and participants were not under

any time pressure, so our reaction time measures should be viewed with caution.

Page 8, lines 144-148:

"Once an aim was recorded, the target turned from orange to green, the aiming ring disappeared, and

the subject was able to begin their reach with the right hand (see Figure 2). Subjects were instructed to

proceed through the experiment as quickly as possible, however, there was no time limit for tapping the

aim location or beginning the reaching movement.”

Line 164-170: the target locations are not obvious for the mirror.

An additional panel was added to the methods figure (Figure 2) in order to more clearly illustrate the

target locations.

The intuition behind the 2 experiments is not obvious by just reading the methods. The first paragraphs of

the results for each exp (line 223 and 309) are helpful and could be presented earlier.

We added this summary at the beginning of the Methods section on page 13, lines 249-251:18

"In two experiments, twenty-four subjects participated in a visuomotor rotation task for one hour each

day for five consecutive days. Subjects attempted to compensate for a visual perturbation of 45{degree sign} while

reaching to targets that appeared on a circle centered on the start-position.
